# Synthesis, Structure, and Photophysical Properties of Platinum Compounds with Thiophene-Derived Cyclohexyl Diimine Ligands

**DOI:** 10.1021/acsomega.3c05567

**Published:** 2023-10-06

**Authors:** Matthew W. Greenberg, Kris M. Tulloch, Michelle E. Reynoso, Juliette L. Knapp, Farman H. Sayem, Daphne D. Bartkus, Ryan H. Lum, Christopher N. LaFratta, Joseph M. Tanski, Craig M. Anderson

**Affiliations:** † Department of Chemistry & Biochemistry, Bard College, 30 Campus Road,Annandale-on-Hudson, New York 12504, United States; ‡ Department of Chemistry, Vassar College, Poughkeepsie, New York 12604, United States

## Abstract

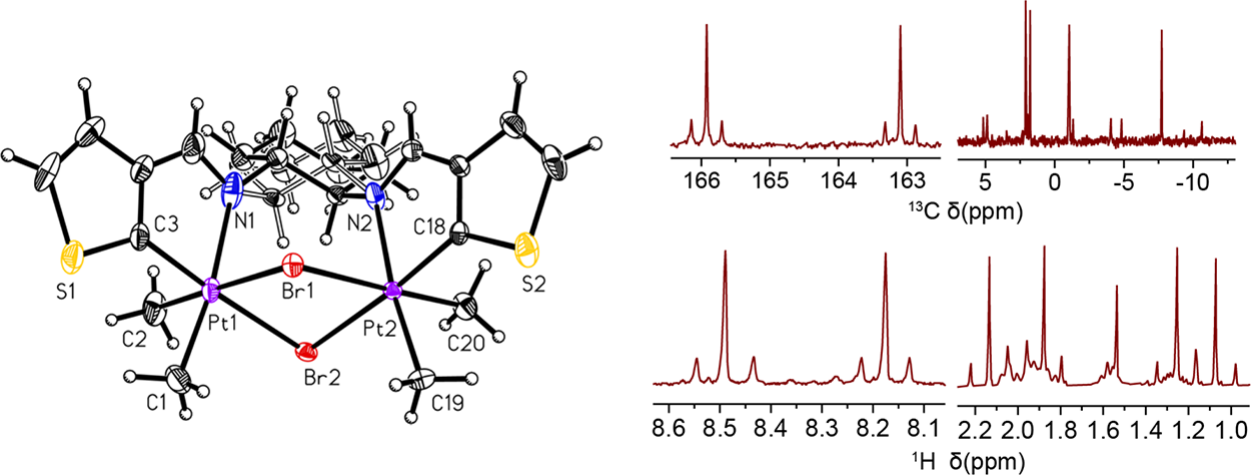

Platinum(II) and platinum(IV) compounds were prepared by the stereoselective and regioselective reactions of thiophene-derived cyclohexyl diimine C^N^N-ligands with [Pt_2_Me_4_(μ-SMe_2_)_2_]. Newly synthesized ligands were characterized by NMR spectroscopy and elemental analysis, and Pt(II)/Pt(IV) compounds were characterized by NMR spectroscopy, elemental analysis, high-resolution mass spectrometry, and single-crystal X-ray diffraction. UV–vis absorbance and photoluminescence measurements were performed on newly synthesized complexes, as well as structurally related Pt(II)/Pt(IV) compounds with benzene-derived cyclohexyl diimine ligands, in dichloromethane solution, as solids, and as 5% by weight PMMA-doped films. DFT and TD-DFT calculations were performed, and the results were compared with the observed spectroscopic properties of the newly synthesized complexes. X-ray total scattering measurements and real space pair distribution function analysis were performed on the synthesized complexes to examine the local- and intermediate-range atomic structures of the emissive solid states.

## Introduction

Platinum(II) and platinum(IV) cyclometalated complexes are an important class of organometallic compounds with varied applications in catalysis, inorganic photophysics, and bioinorganic chemistry.^1−4^ Cyclometalated ligands featuring chelating- or pincer-type binding to the central Pt atom with a strong carbanionic σ-donor are especially well studied in this regard.^5,6^ In the context of luminescent complexes, these strong field ligands are known to raise the energy of unoccupied metal-centered (MC) states that could otherwise provide nonradiative pathways to the deactivation of the excited state.^7^ Among cyclometalated Pt compounds, square-planar Pt(II) *d*^8^ complexes are the most widely explored for luminescent complexes with long-lived excited states aided by fast intersystem crossing rates.^3,8−10^ In comparison, octahedral Pt(IV) complexes possess lower energy 5d orbitals that can be expected to have poorer mixing with ligand orbitals, resulting in Pt(IV) compounds being far less studied than Pt(II) compounds as emissive complexes.^11^ However, the potential of octahedral *d*^6^ cyclometalated Pt(IV) complexes as emitters has become more appreciated in recent years.^11−13^

Cyclometalated complexes of Pt are most often prepared by the well-known C–H and C–X (X = Cl, Br, I) oxidative addition reactions of appropriately designed ligands. The initial coordination of neutral donor atoms in the ligands such as N, O, and P direct the oxidative addition in heteroatom-assisted C–H/C–X activation, leading to the cyclometalated product.^4,14−16^ In order to realize high-yielding syntheses of cyclometalated Pt complexes, ligands that react with high regioselectivity and stereoselectivity are essential. In general, this requires careful ligand design, as many possible regiochemical and stereochemical products may result from C–H/C–X reactivity of organic ligands. Schiff base ligands with an imine nitrogen donor atom are a commonly used class of ligands for the purposes of cyclometalation due to their ease of synthesis and tendency to metalate to form (most commonly) five- and six-membered cyclometalated rings.^5,6,17,18^ In one example, Puddephatt and co-workers reported cyclohexyl diimine C^N^N ligands derived from chiral cyclohexyl diamine and benzaldehyde that react to form cyclometalated complexes with very high selectivity for both Pt(II) and Pt(IV) complexes.^19,20^ Furthermore, cyclometalated C^N^N ligands are known to form luminescent Pt(II) and Pt(IV) complexes.^11,21,22^

**Scheme 1:**
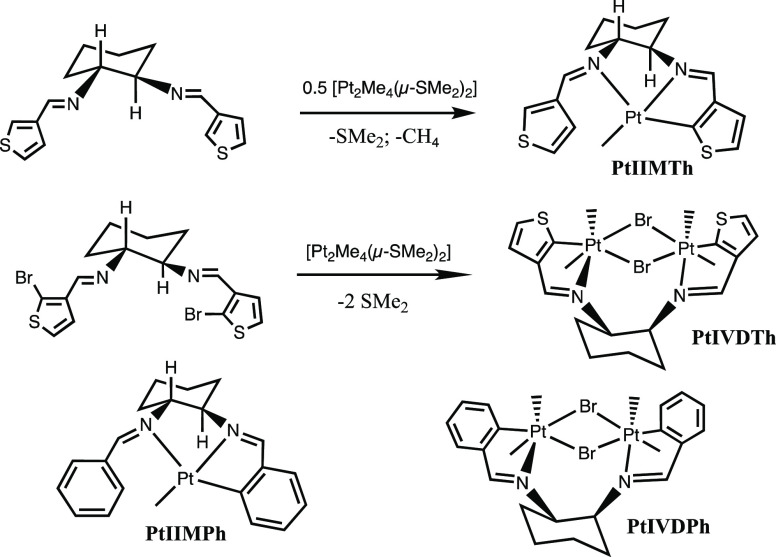
Syntheses of New Platinum Compounds, **PtIIMTh** and **PtIVDTh**, and the Structures of the Benzene Analogues, **PtIIMPh** and **PtIVDPh**

In this work, we explore a new type of cyclohexyl diimine C^N^N ligand featuring a thiophene ring, as is shown in Scheme 1. Conjugated thiophene polymers and oligomers are attractive organic optoelectronic materials in their own right,^23−25^ and metal complexes featuring metalated or pendant thiophenes with interesting photophysical properties have been reported.^26−29^ In particular, metalated thiophenes as compared to phenyl groups in structurally analogous Pt(II) complexes have been reported to enhance fluorescence over phosphorescence in recent studies.^26,30^ Among aromatic rings, thiophenes have been reported to be reactive toward C–H activation and are excellent candidates for preparing cyclometated complexes through C–H activation reactions.^14,31^ Using this diimine C^N^N ligand framework, we report the synthesis, structural characterization, and photophysical properties of new Pt(II)/Pt(IV) complexes and have compared their properties with the structurally related benzene-derived complexes reported earlier by Puddephatt.^19,20^ We report their luminescence in both solution and solid media for these four complexes, as a major focus of recent research in luminescent inorganic complexes is the impact of physical state and intermolecular order on photophysical properties.^32−35^

## Results and Discussion

### Synthesis and Characterization

The new thiophene-derived diimine ligands were synthesized by condensation reactions, as described in the Experimental Section, and their NMR spectra are included in the Supporting Information (Figures S1–S6). The ligands were characterized by multinuclear NMR spectroscopy, IR spectroscopy, and elemental analysis. The ligands were used to synthesize two platinum compounds by reacting them with the tetramethyl platinum dimer (Scheme 1). The platinum compounds were characterized by multinuclear NMR spectroscopy (Figures S7–S16), IR spectroscopy, elemental analysis, high-resolution mass spectrometry, and single-crystal X-ray diffraction techniques. Ligand **A** gave a platinum(II) species, **PtIIMTh**, formed by C–H activation, followed by methane elimination. Ligand **B** gave a platinum(IV) dimer, **PtIVDTh**, formed by intramolecular C–Br oxidative addition. These reactions were accompanied by a high degree of stereoselectivity for the isolated products, comparable to analogous species with benzene-derived ligands previously reported.^19,20^ With our isolated yields of close to 70%, only a single diastereomer is observed. The previously reported platinum compounds could be synthesized by heating and stirring as well as by utilizing a microwave reactor. Similar results were observed in either synthetic case.

**Figure 1:**
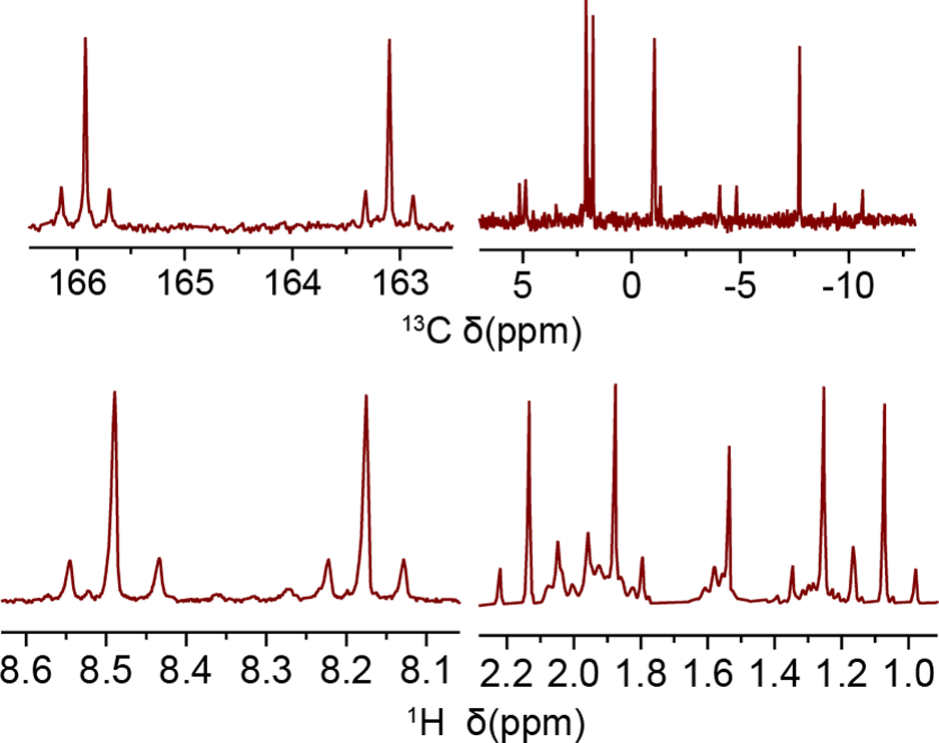
^13^C{^1^H} (top) and ^1^H (bottom) NMR spectra of imine and methyl resonance regions for **PtIVDTh** containing ^195^Pt–^13^C/^1^H coupling.

The proton spectrum of **PtIIMTh** contains two imine resonance and one Pt-Me resonance, as expected for a monomeric species with an N^N^C ligand and one methyl ligand in the coordination sphere (Scheme 1). Each of the platinum(IV) species’ ^1^H and ^13^C NMR spectra has four methyl resonances and two imine resonances (Figure 1). The *J*(Pt–H) and *J*(Pt–C) coupling constants observed for these new complexes are consistent with the literature values for Pt(II) and Pt(IV) complexes.^19,20,36−38^ The formation of the compounds was indicated in the infrared spectra by a frequency shift of the C=N bond stretching from the free ligand. For **PtIVDTh,** this band shifted from 1628 to 1582 cm^–1^, and for **PtIIMTh,** it shifted from a single band at 1636 to two separate bands at 1621 and 1587 cm^–1^ due to the existence of one metalated thiophene ring and one dangling thiophene ring (Figures S17–S20). This was corroborated with the DFT-calculated IR spectra (Figures S21 and S22), where visualization of the normal modes shows a higher energy C=N stretch for the imine attached to the dangling thiophene and a lower energy C=N stretch for the imine contained within the cyclometalated ring.

**Figure 2:**
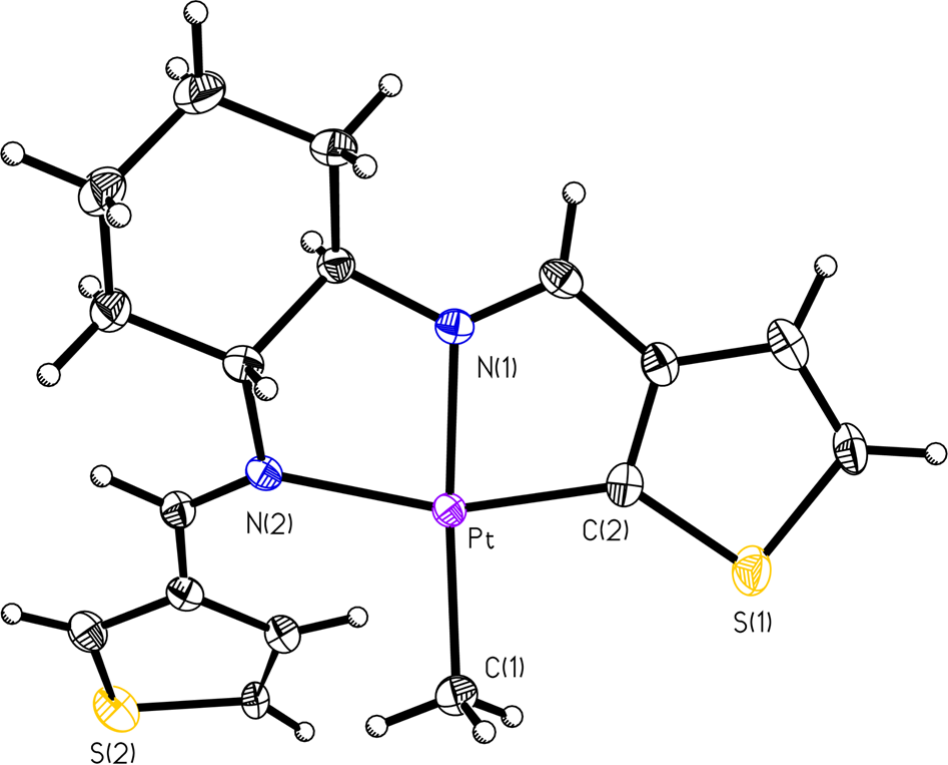
ORTEP of Compound **PtIIMTh** (50% probability thermal ellipsoids). Selected bond lengths (Å) and angles (deg): Pt–C2:1.967(2); Pt–N1:2.045(2); Pt–C1:2.054(3); Pt–N2:2.098(2); C2–Pt–N1:79.85(10); C2–Pt–C1: 96.00(11); N1–Pt–C1:171.12(10); C2–Pt–N2; 160.17(9); N1–Pt–N2:80.32(8); C1–Pt–N2:103.55(9).

**Figure 3:**
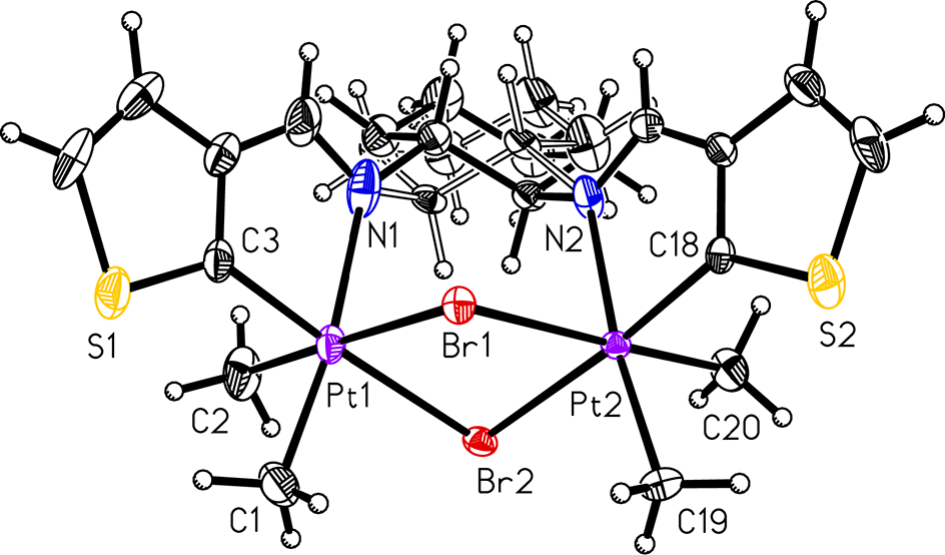
ORTEP of Compound **PtIVDTh** (50% probability thermal ellipsoids). Selected bond lengths (Å) and angles (deg): Pt1–C3:1.968(4); Pt1–C1:2.054(4); Pt1–C2:2.057(4); Pt1–N1:2.233(4); Pt1–Br2:2.5778(4); Pt1–Br1:2.6104(4); Pt2–C18:1.960(3); Pt2–C20:2.052(3); Pt2–C19:2.060(4); Pt2–N2:2.173(3); Pt2–Br2:2.5777(4); Pt2–Br1:2.6273(4); C3–Pt1–C1:93.81(16); C3–Pt1–C2:91.46(16); C1–Pt1–C2:87.5(2); C3–Pt1–N1:78.05(14); C1–Pt1–N1:171.77(14); C2–Pt1–N1:91.49(17); C3–Pt1–Br2:175.90(11); C1–Pt1–Br2:88.26(12); C2–Pt1–Br2:92.17(12); N1–Pt1–Br2:99.94(9); C3–Pt1–Br1:90.20(11); C1–Pt1–Br1:92.93(13); C2–Pt1–Br1:178.24(13); N1–Pt1–Br1:88.29(9); Br2–Pt1–Br1:86.145(13); C18–Pt2–C20:86.33(14); C18–Pt2–C19:93.98(15); C20–Pt2–C19:89.53(16); C18–Pt2–N2:79.07(12); C20–Pt2–N2:88.35(14); C19–Pt2–N2:172.84(14); C18–Pt2–Br2:174.97(9); C20–Pt2–Br2:94.41(11); C19–Pt2–Br2:91.00(11); N2–Pt2–Br2:95.98(8); C18–Pt2–Br1:93.32(9); C20–Pt2–Br1:178.37(12); C19–Pt2–Br1:92.09(11); N2–Pt2–Br1:90.02(8); Br2–Pt2–Br1:85.797(13); Pt1–Br1–Pt2:89.243(13); Pt2–Br2–Pt1:91.061(14).

The two new species were also characterized by single-crystal X-ray diffraction techniques (Figures 2 and 3), and the structures confirm those predicted by NMR spectroscopy, as illustrated in Scheme 1. **PtIIMTh** (Figure 2) has a tridentate pincer ligand with a dangling thiophene ring and one methyl ligand. The Pt–C and Pt–N bond lengths and the bond angles around the platinum center are within acceptable ranges for the platinum(II) species.^6^**PtIVDTh** (Figure 3) does not form a monomeric species with the C^N^N ligand but instead forms a dimeric species with one ligand unit bridging two platinum centers with C^N attachments on each metal center. Two bromide ligands, formed by intramolecular oxidative addition, bridge the platinum atoms, and each platinum center also has two methyl ligands. Once again, the bond lengths and angles are within acceptable ranges for platinum(IV) species with C^N chelate and bromide bridging ligands.^13,19,20^

**Figure 4:**
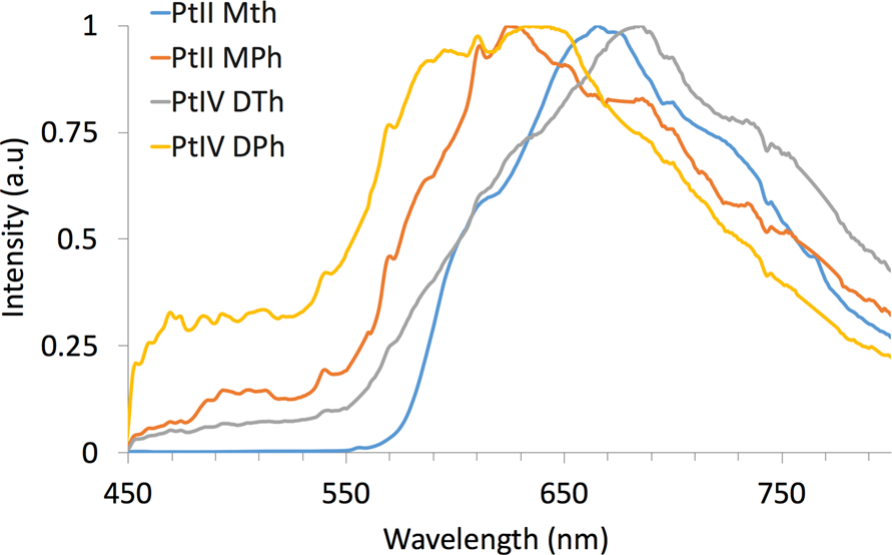
Solid state emission spectra of platinum compounds excited at 400 nm. Raman spikes have been removed. See Figures S24 and S25 for reference.

The photophysical properties of the compounds were probed to aid in the characterization of the species and compared to those of the benzene-derived compounds. The absorbance spectra (Table S1 and Figure S23) for the platinum(II) species show their lowest energy peaks in the 400–500 nm range, with extinction coefficients of 10^3^ thus tentatively assigned to MLCT transitions (see below for further assignments with the aid of TD-DFT). The platinum(IV) complexes (Table S1 and Figure S23) show their lowest energy band in the UV region, centered at a higher energy at around 350 nm, as expected for the higher oxidation state.^11,13^ The steady-state emission spectra and lifetime measurements for all four species were conducted for solid compounds, for samples in dichloromethane (DCM) solution, and for 5% by weight doped PMMA films (Table S2 and Figures S24–S29). The emission spectra in DCM solution show wide spectral bands of approximately 200–250 nm with subtly defined multiple and/or shoulder peaks. The samples in solution had low quantum yields of ≤0.1% for all species. The spectra of the solid state samples (Figure 4) also show large spectral width but are slightly red-shifted and are essentially one large, broad band with fewer defined shoulders. The solid state samples also had low quantum yields but greater than those in solution for the platinum(II) species. The quantum yield of **PtIIMTh** in the solid state was determined to be 1.8%, and for **PtIIMPh,** it was determined to be 0.8%. Values could not be obtained for the two Pt(IV) solid samples given the limitation of the integration sphere for quantum yields below 0.8%. The steady-state spectra of the 5% doped films of the two Pt(II) species and **PtIVDTh** were somewhat similar. They consist of broad bands, with the emission maxima centered around 610–650 nm. In contrast, **PtIVDPh** emits at a lower wavelength than the other three species and has several multiple/shoulder peaks. In contrast to the solid state samples, the quantum yields of the Pt(II) species as PMMA-doped films did not give a measurable quantum yield in the integration sphere due to the sphere’s limitations at low quantum yield and thus presumed to be less than 0.8%; however, measurable results were obtained for the two platinum(IV) PMMA-doped samples. The quantum yield of **PtIVDPh** was determined to be 2.6% and that of P**tIVDTh** was determined to be 1.0%. The Pt(IV) species’ quantum yield values were the greatest in the rigid PMMA medium; thus, these samples can be said to exhibit rigidoluminescence.^39^ Conversely, the Pt(II) species’ values were best in solid state samples, exhibiting an enhancement compared to those of their samples in other states. The lifetimes of emission peaks in both the solid state and in DCM solution (Table S3) are in the hundred nanosecond range and would tentatively indicate an excited state with mixed ^3^MLCT/^3^LC character (see below). The lifetime values obtained for the PMMA films were also in the hundreds of nanosecond range. Additionally, all samples also had a second, much shorter lived state in the range 12–23 ns, as the decay curves best fit a double exponential decay (Table S3 and Figure S38). We suggest a possible fluorescence for the faster lifetime.^26,29,31^

### TD-DFT and DFT Calculations

**Figure 5:**
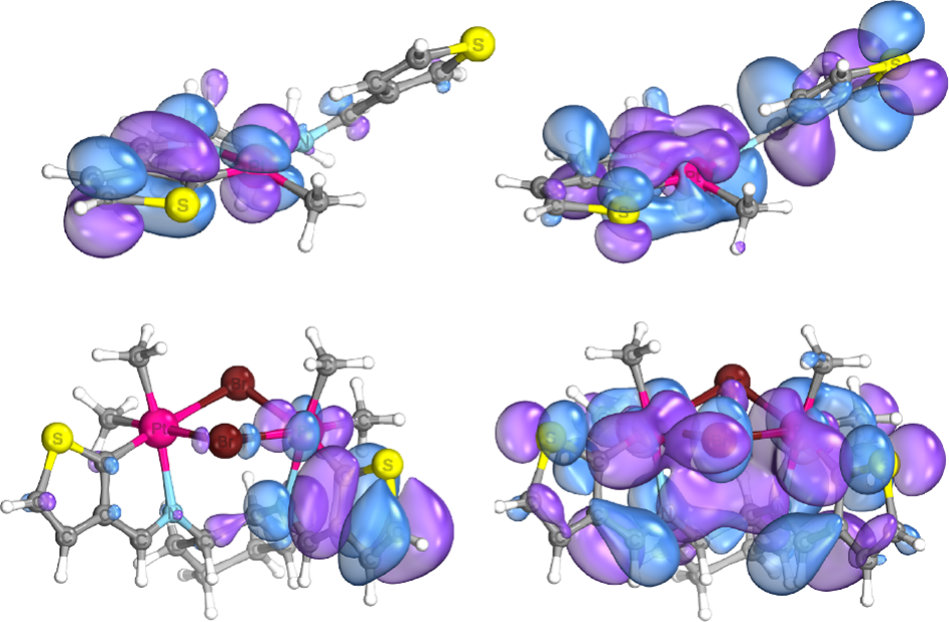
DFT-calculated HOMO (left) and LUMO (right) for **PtIIMTh** (top) and **PtIVDTh** (bottom). Orbitals are visualized using an 80% isosurface threshold using the IboView program.^41^

Density functional theory (DFT) and time-dependent DFT (TD-DFT) calculations were run on the Pt(II) and Pt(IV) complexes and compared with the observed electronic transitions measured by UV–vis absorbance spectroscopy. As mentioned previously, the large extinction coefficients observed for the UV–vis absorbance features are consistent with MLCT, as has been assigned in structurally similar Pt(II) and Pt(IV) C^N^N, C^N cyclometalated complexes.^13,31,40^ The DFT-calculated HOMO and LUMO of **PtIIMTh** and **PtIVDTh** are shown below. In each case, the HOMO is spread across the π orbitals of the thiophene ligand and Pt *d* orbitals, while the LUMO is more clearly of π* character across the ligand. The near-frontier orbitals below the HOMO and above the LUMO are also plotted in Supporting Information (Figures S39–S40 and S43–S44) and are similarly distributed across π/π* systems and Pt metal orbitals. The results are analogous in the case of the phenyl diimine ligand Pt(II) and Pt(IV) complexes, except with the π/π* orbitals of the phenyl group, as shown in Figures S41, S42, S45, and S46. The two aromatic rings of the diimine ligand are electronically inequivalent in both Pt(II) and Pt(IV) complexes, which can be clearly seen in the HOMOs of both complexes, as shown in Figure 5.

**Figure 6:**
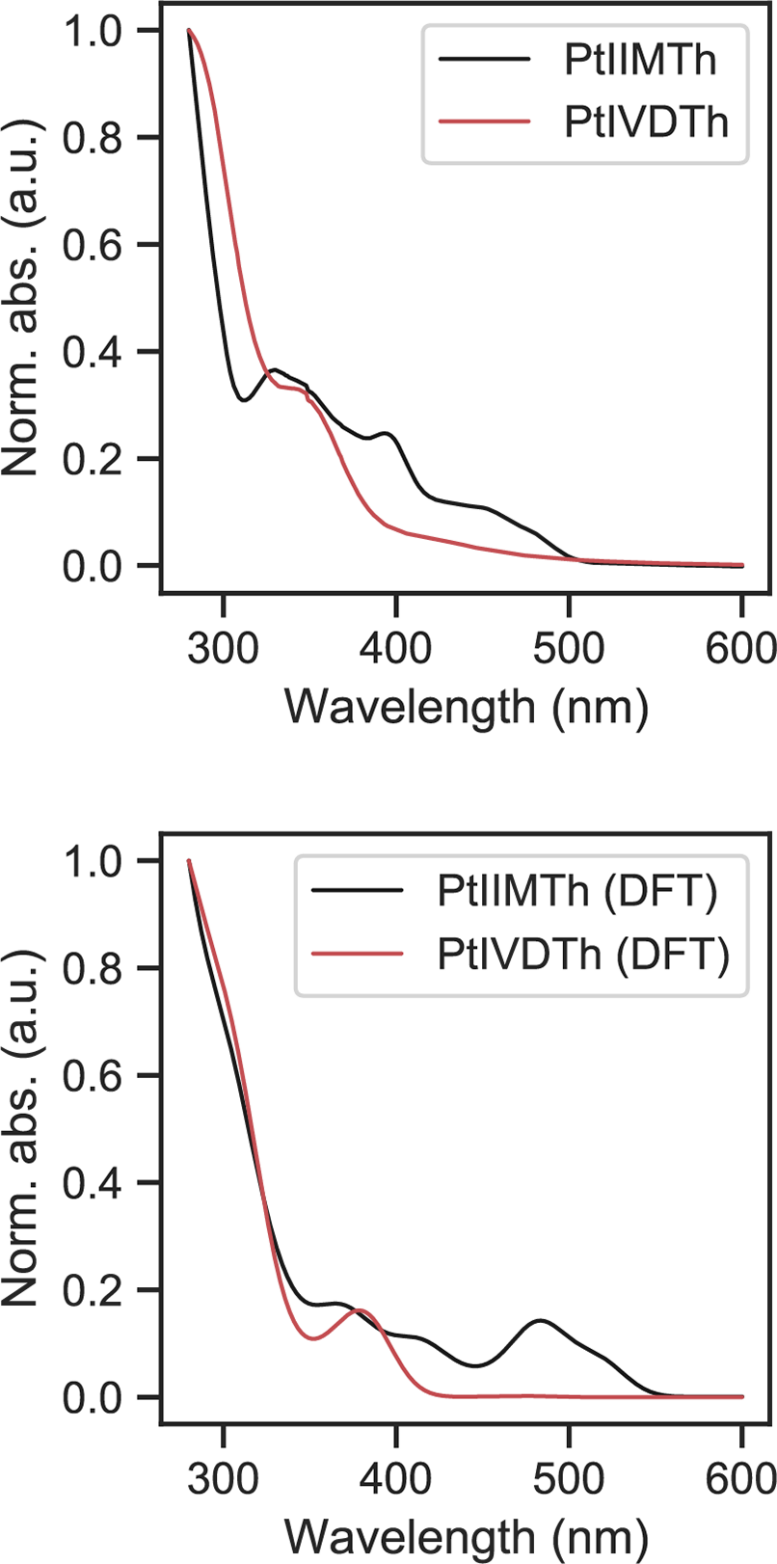
(Top) Observed normalized absorbance spectrum of **PtIIMTh** and **PtIVDTh** and TD-DFT-calculated normalized absorbance spectrum. The band shape for the TD-DFT-simulated absorbance spectrum is obtained by broadening the vertical excitations with a 30 nm linewidth (Figures S47 and S48) and then normalizing the oscillator strength.

The TD-DFT-simulated absorbance spectrum is shown and compared with the normalized experimental UV–vis spectra in Figure 6. A good qualitative agreement in transition intensities and energies with the experimental data for this level of theory was seen for both Pt(II) and Pt(IV) complexes. The full set of all calculated vertical excitations is shown for all compounds in Figures S36–39. The expected relative blue shift of Pt(IV) versus Pt(II) lowest energy UV–vis absorbance features involving the metal *d* orbitals is clearly reproduced in these calculations. We conclude that the observed experimental UV–vis absorbance is consistent with the DFT level of theory for the novel compounds, further confirming their identity. Experimental and DFT theory evidence suggest that the UV–vis electronic transitions possess MLCT/LLCT (ligand-to-ligand charge transfer) character, as frontier and near-frontier orbitals are unevenly distributed between the π systems of the two inequivalent aromatic rings in each complex.

### Real-Space X-ray Pair Distribution Function Analysis

Enhanced emission quantum yields and/or distinct emission profiles in the solid state compared to the solution state for transition-metal complexes are often observed. In many cases, this is believed to be related to the structural coherence as well as the rigidity of the solid state.^33^ While laboratory powder X-ray diffraction measurements are often performed in these studies and can be used to identify crystalline phases, standard powder diffraction experiments provide limited information regarding amorphous or disordered/nanocrystalline atomic structures.^42^ Many emissive solid samples of transition-metal complexes obtained from solution synthesis are not crystalline, and the role of structural coherence in aggregation-induced emission for molecular solids has been a focus of several recent reports.^32,34,35^ A general technique for studying the atomic structure of amorphous, nanocrystalline, and crystalline materials is analysis of the real-space X-ray pair distribution function *G*(*r*). The X-ray pair distribution function *G*(*r*) is a histogram of the interatomic distances in the sample and can be obtained by the Fourier transform of *F*(*Q*), the normalized total scattering structure function obtained from synchrotron X-ray total scattering measurements. The utility of this technique for the structural study of molecular materials has been reviewed recently,^43^ and several reports highlight the applications for local structural studies of inorganic and organometallic molecular solids.^32,44−46^

**Figure 7:**
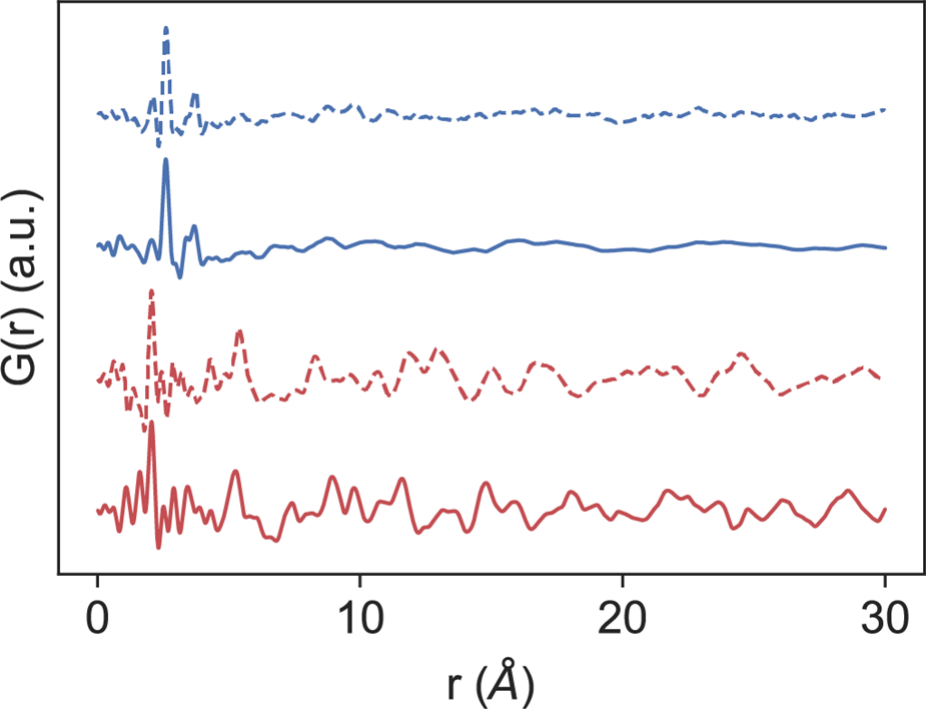
X-ray *G*(*r*) data for **PtIIMTh** (red), **PtIIMPh** (dashed red), **PtIVDTh** (blue), and **PtIVDPh** (dashed blue).

X-ray *G*(*r*) spectra were recorded in the solid state for **PtIIMTh, PtIIMPh, PtIVDTh**, and **PtIVDPh,** as illustrated in Figure 7, with the reciprocal space *F*(*Q*) plotted in Figure S40. The Pt(II) and Pt(IV) complexes for thiophene versus benzene show very similar local- and intermediate-range structures by the inspection of *G*(*r*), which are consistent with the similar molecular structures of the benzene versus thiophene analogues. The Pt(II) complexes show their most intense correlation at around 2.05 Å, which is consistent with the overlap of the Pt–C/Pt–N coordination sphere distances listed in the caption of Figure 2 found in our single-crystal X-ray diffraction study. The Pt(IV) complexes on the other hand show the most intense correlation at around 2.60 Å, with a smaller broad first peak around 2.10 Å. In the case of the Pt(IV) complexes, the most intense correlation can be assigned to the Pt–Br distance whose intensity can be explained by (1) the relatively large atomic form factors of the Pt–Br pair and (2) the presence of four Pt–Br distances per molecule in the dimeric structure. The smaller first correlation centered around 2.10 can again be assigned to the overlapped Pt–C/Pt–N distances and is consistent with the distances listed in the caption of Figure 3. Simulation of the PDF using the Debye scattering equation and accompanying partial PDFs for both single molecules and with the local intermolecular structure from the packing of the single-crystal unit cell generated in Mercury^47^ corroborate the assignments discussed above and are shown in Figures S54 and S55.

**Figure 8:**
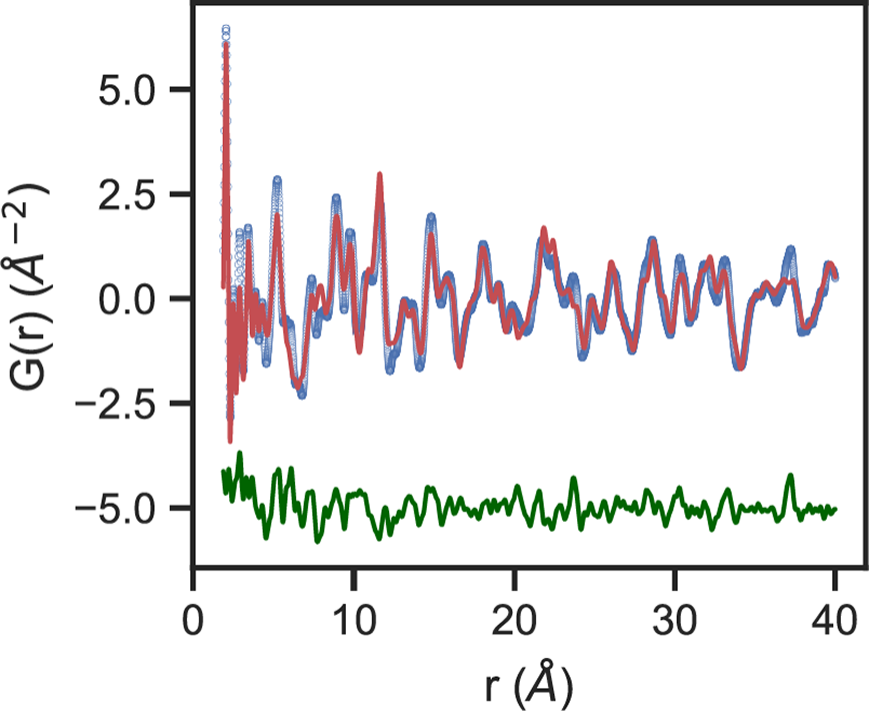
*G*(*r*) data (blue), fit (red), and difference (green) from *r* = 1.9 to 40 Å for a virtual crystal model based on the single-crystal intermolecular packing for **PtIIMTh**.

Beyond the local structure in *G*(*r*) and extending into the intermolecular distance (*r* > 5 Å) ranges, the Pt(II) and Pt(IV) complexes with thiophene versus benzene rings show good qualitative agreement with each other. The Pt(II) complexes show extended atomic order past the intramolecular distances, consistent with a high degree of intermolecular order in the solid state. The Pt(IV) complexes also show evidence of intermolecular order in their solid states; however, the absence of sharp peaks past 1 nm is consistent with a more disordered solid state as compared to the Pt(II) complexes. The flat square-planar geometry at Pt(II) may more easily template a close intermolecular packing, as shown in Figure S52 for the crystalline solid state, as compared to the Pt(IV) complexes shown in Figure S53, which are seen cocrystallized with CHCl_3_ solvent molecules in our single-crystal structure. The observed *G*(*r*) for **PtIIMTh** can be modeled using an established method for treating *G*(*r*) of molecular materials that accounts for separate inter- and intramolecular ADPs and a finite crystal coherence length.^43,48−51^ The fit (*r*_w_ = 0.32) is shown in Figure 8, while the fit parameters are listed in Table S4 alongside a description of the model. While the virtual crystal model from the single-crystal structure does not fully account for the as-synthesized solid state data, the relatively good agreement in capturing both inter- and intramolecular distance ranges suggests a similar packing of molecules in the bulk synthesized solid sample to the single-crystal solid state.

## Concluding Remarks

Thiophene-derived C^N^N diimine ligands were used to synthesize new Pt(II) and Pt(IV) cyclometalated complexes with high reaction selectivity for the isolated products. These products were characterized by multinuclear and 2D NMR spectroscopy, and their atomic structures were determined by single-crystal X-ray diffraction. UV–vis absorbance and photoluminescence measurements of these compounds were performed and compared to those of previously reported compounds synthesized with analogous benzene-derived diimine ligands. The results were compared with the DFT and TD-DFT calculations. The newly synthesized solid sample of **PtIIMTh** showed the highest recorded quantum yield of the surveyed complexes. The structural order present in the emissive solid state was examined for these complexes by X-ray pair distribution function analysis, with the Pt(II) complexes showing clear evidence of intermolecular ordering. Collectively, these results demonstrate the use of these thiophene-derived C^N^N diimine ligands to selectively prepare photoactive cyclometalated transition-metal complexes with high chemical selectivity.

## Experimental Section

### General

Solvents and reagents were purchased from Sigma-Aldrich unless otherwise noted. K_2_PtCl_4_ was purchased from J. and J. Materials (NJ). NMR spectra were recorded at Bard College using a Varian NMR-400 MHz spectrometer (^1^H, 400 MHz; ^13^C, 100.6 MHz). Shifts are given in parts per million and coupling constant *J* values in Hertz. Abbreviations used: s = singlet; d = doublet; t = triplet; and m = multiplet. Electrospray MS was performed at Vassar College using an Agilent LC/MSD-TOF spectrometer. Samples were run in chloroform in negative mode.

### Computational Details

TD-DFT and DFT calculations were performed using Orca ab initio quantum chemistry program version 4.2.1.^52,53^ All calculations were performed using the B3LYP functional.^54,55^ The LANL2DZ basis set with an effective core potential used for heavy atoms (Pt, S, and Br) was used during initial geometry optimization and vibrational frequency calculations.^56^ All final structures were confirmed to be at an energetic minimum by harmonic vibrational analysis. TD-DFT calculations using the Tamm–Dancoff Approximation were performed as single-point calculations, with optimized structures at the ZORA-def2-TZVP level of theory, with an auxiliary basis of SARC/J-ZORA-TZVP for Pt and a CPCM solvation model for the DCM solvent.^57^ The UV–vis excitations were calculated with the first 30 singlet and triplet excited states and spin–orbit coupling. Theoretical vertical excitations are broadened with a 30 nm linewidth for the purposes of visualization of the band shapes in Figure 6. Initial geometry guesses were generated by using crystallographic data.

### Photophysical Measurements

Steady-state emission spectra were recorded by using a PTI QM-40 instrument with a PMT detector, which is sensitive up to 850 nm. In these experiments, the concentration of the platinum complexes ranged from 22 to 41 μM. The fluorimeter emission spectrum was corrected using a method described in the literature which uses four standard fluorophores to calibrate the response of the instrument.^58^ The slits were set at 2.5 nm bandpass for all solution measurements. Long-pass filters were used to block the excitation light and avoid the detection of its second harmonic. Solid state samples were sandwiched between two pieces of 1 mm thick borosilicate glass slides and mounted at 45° such that the detected luminescence was from the back powder and the specular reflection pointed away from the detector. The slits were set at 1.25 nm bandpass for the solid state samples. The luminescence lifetimes of the complexes were measured by time-correlated single-photon counting (TCSPC) following excitation with a 365, 405, or 450 nm LED. For TCSPC measurements, the slits were adjusted such that <3% of the LED flashes resulted in a detection event ensuring such events are single photons. Solution samples were degassed for 5 min prior to measurement. The 5% by weight doped PMMA films were made by weighing approximately 1 mg of compound and dissolving it in 0.5 mL of HPLC-grade DCM. An appropriate amount of PMMA (poly(methyl methacrylate-*co*-butyl methacrylate), average *M*_w_ ∼ 75 kDa) was weighed to give a weight ratio of 5:95 and dissolved in the DCM solution. After several minutes of stirring, the solution was poured onto a Teflon block and allowed to dry overnight. The films were approximately 1 cm^2^ in area. The films were mounted at 45°, like the solid state samples, for the luminescence measurements. Quantum yield measurements for solution samples were prepared by serial dilution, all having absorbance values <0.1, and their absorbance and integrated fluorescence signals were compared to aqueous [Ru(bpy)_3_]Cl_2_ solutions having similar absorbances. The solid state samples were placed in a Teflon powder holder mounted within a petite integrating sphere (Horiba, *K*-sphere). During quantum yield measurements, the slit sizes were adjusted, and a neutral density filter with an optical density of 1.0 was used to keep the signal in the linear range of the detector (<1 Mcps). Excitation and emission slits were typically set to ∼1 nm bandpass. BaSO_4_ was used as the reference compound for the solid state samples, and an undoped PMMA film was used as the reference for the doped PMMA samples. The accuracy of the integrating sphere was checked using Rhodamine 101 in ethanol and also using [Ru(bpy)_3_]Cl_2_ in water as standards, with known quantum yields from the literature of 0.92 and 0.042, respectively.^59−61^ The measured values were found to be 0.93 and 0.041, respectively.

### X-ray Diffraction

**PtIIMth** was crystallized by the slow diffusion of pentane into an acetone solution, while **PtIVDTh** was crystallized by the slow diffusion of pentane into a chloroform solution. X-ray diffraction data were collected on a Bruker APEX 2 CCD platform diffractometer (Mo Kα (*l* = 0.71073 Å)) at 125 K, with the crystals mounted on a nylon loop with Paratone-N cryoprotectant oil. The structures were solved using direct methods (SHELXT 2018/2)^62^ and standard difference map techniques and were refined by full-matrix least-squares procedures on F2 (SHELXL 2017/1).^63^ In **PtIVDTh**, a two-fold disorder of the cyclohexyl fragment was modeled and refined (FVAR 0.677(9)) with the help of similarity restraints on bond lengths and angles as well as on displacement parameters. In **PtIIMth**, a slight (∼10%) two-fold rotational disorder in the S2-containing thiophene did not model sufficiently well and was left unmodeled. All nonhydrogen atoms were refined anisotropically.

### X-ray Total Scattering Measurements and Pair Distribution Function Analysis Data Reduction

Total X-ray scattering measurements were performed at Brookhaven National Laboratory using the 28-ID-2 (XPD) high-energy X-ray powder diffraction beamline at the National Synchrotron Light Source II (NSLS-II). X-ray scattering data were collected using a large-area 2D PerkinElmer detector (2048 × 2048 pixels, 200 × 200 μm^2^ each) in RA-PDF mode with a sample-to-detector distance of 224 mm.^64^ The incident energy of the X-rays was 67.13 keV (λ = 0.1847 Å). Solid samples were loaded into 1.5 mm Kapton polyimide tubes, and the capillaries were sealed with modeling clay. Beamline calibration was performed with a Ni powder standard. 2D detector intensity images were azimuthally integrated using PyFAI to 1D *I*(*Q*) curves, where  is the magnitude of the elastic scattering momentum transfer.^65^ Subtraction of the sample holder background, polarization correction, normalization to the reduced total scattering structure function *F*(*Q*), and Fourier transformation to obtain the pair distribution function *G*(*r*) were carried out using PDFgetX3 implemented in xPDFsuite.^66,67^ The range of scattering vectors used in the Fourier transform to obtain *G*(*r*) was chosen to optimize the trade-off between real-space resolution and statistical noise (*Q*_max_ = 18 Å^–1^, Figure S40). PDF simulations of *G*(*r*) were performed using custom code written in Python utilizing PDF calculators in the DiffPy-CMI complex modeling^68^ framework, and structural refinement was performed in PDFGui,^69^ and the refined parameters and output are listed in Table S4.

### Preparation of Compounds

See the Supporting Information for additional experimental details, including copies of NMR spectra, UV/vis spectra, and emission spectra. [Pt_2_Me_4_(μ-SMe_2_)_2_] was prepared according to the literature.^70^**PtIIMPh** and **PtIVDPh** were synthesized according to the literature^19,20^ and using a microwave reactor. For example, 0.260 g of [Pt_2_Me_4_(μ-SMe_2_)_2_] and 0.0391g of racemic *trans*-1,2-(N=CHC_4_H_2_SBr)_2_C_6_H_10_ were dissolved in 10 mL of acetone in a CEM microwave reactor vial. The microwave was set at 40 °C and 100 psi for 30 min. The solvent was removed under vacuum, and the resulting solid was triturated with several 1.5 mL portions of diethyl ether before being dried under vacuum. This afforded a gray product (Yield, 0.0303 g, 74.5%).

### Ligand A

#### Racemic *trans*-1,2-(N=CHC_4_H_3_S)_2_C_6_H_10_

To a solution of 3-thiophenecarboxaldehyde (891 mg, 8.0 mmol) in DCM (20 mL), racemic trans-1,2,-diaminocyclohexane (465 mg, 4.07 mmol) was added. Excess magnesium sulfate was added to remove water formed during the reaction. The reaction was stirred at room temperature for 6 h. MgSO_4_ was removed by gravity filtration. The solvent was removed under vacuum, resulting in a light brown solid. Yield: 62%. ^1^H NMR (400 MHz, CDCl_3_): δ = 1.47 [m, 2H, Cy(H)], 1.76 [m, 2H, Cy(H)], 1.84 [m, 4H, Cy(H)], 3.30 [m, 2H, Cy(H)], 7.22 [dd, 2H], 7.40 [dd, 2H], 7.45 [dd, 2H], 8.18 [s, 2H, N=CH]. ^13^C NMR (100 MHz, CDCl_3_) δ = 24.6, 33.1, 73.9, 125.9, 126.1, 127.9, 140.7, 155.5. Elemental analysis, % calculated for C_16_H_18_N_2_S_2_: C, 63.54; H, 6.00; N, 9.26; Found: C, 62.23; H, 5.72; N, 9.00.

### Ligand B

#### Racemic *trans*-1,2-(N=CHC_4_H_2_SBr)_2_C_6_H_10_

To a solution of racemic trans-1,2-diaminocyclohexane (0.184 g) in diethyl ether (10 mL), 2-bromothiophene-3-carbaldehyde (0.366 g) was added. 1 mL of 3 Å molecular sieves was added to remove water formed during the reaction. The mixture was stirred at room temperature for 14 h. The ether was removed by evaporation. 15 mL of DCM was added to the residue with the sieves and brought to a boil. The mixture was filtered while hot by gravity to remove the sieves. The resulting solution was placed in a −20 °C freezer for 24 h. The resulting white solid was collected with a Hirsch funnel and dried under vacuum. Yield: 37% (0.182 g). ^1^H NMR (400 MHz, CDCl_3_): δ = 1.48 [m, 2H, Cy(H)], 1.79, 1.85 [m, 6H, Cy(H)], 3.36 [m, 2H, Cy(H)], 7.15 [d, 2H], 7.33 [d, 2H], 8.16 [s, 2H, N=CH]. ^13^C NMR (100 MHz, CDCl_3_) δ = 24.6, 33.0, 73.8, 116.7, 126.0, 126.6, 137.8, 154.6. Elemental analysis, % calculated for C_16_H_16_Br_2_N_2_S_2_: C, 41.76; H, 3.50; N, 6.09; Found: C, 41.66; H, 3.40; N, 5.94.

### PtIIMth

To a solution of ligand **A** (74 mg) in diethyl ether, [Pt_2_Me_4_(μ-SMe_2_)_2_] (70 mg) was added. Within minutes, a bright orange precipitate formed. The mixture was stirred for 14 h, and then the solvent was removed under vacuum. The resulting solid was recrystallized from DCM/pentane. The resulting red solid was isolated and washed with 2 mL of ice cold ether and 3 × 2 mL of pentane and then dried under vacuum. Yield: 73% (91 mg). ^1^H NMR (400 MHz, CDCl_3_): δ = 0.99 [s, 3H, ^2^*J*(PtH) = 83 Hz, Pt-Me], 1.40 [br, m, 2H, Cy(H)], 1.59 [m, 2H, Cy(H)], 2.01 [m, 2H, Cy(H)], 2.41 [d, 1H, Cy(H)], 2.58 [d, 1H, Cy(H)], 3.65 [t, 1H, Cy(H)], 4.21 [t, 1H, Cy(H)], 7.01 [d, 1H, ^4^*J*(PtH) = 32], 7.20 [d, 1H], 7.29 [dd, 1H], 7.95 [d, 1H], 8.45 [s, 1H, ^3^*J*(PtH) = 52 Hz], 8.48 [d, 1H], 8.86 [s, 1H, ^3^*J*(PtH) = 63]. ^13^C NMR (100 MHz, CDCl_3_) δ = −20.8 (CH_3_) ^1^*J*(PtC) = 734 Hz, 24.6, 24.8, 30.0 *J*(PtC) = 12.9 Hz, 30.2, 68.6 *J*(PtC) = 17.4 Hz, 76.0 *J*(PtC) = 25.3 Hz, 97.4, 123.3 *J*(PtC) = 59.7 Hz, 125.6 *J*(PtC) = 58.2 Hz, 128.5, 133.0, 136.7, 149.3, 155.8 *J*(PtC) = 20.0 Hz, 156.5, 157.1 *J*(PtC) = 74.6 Hz. ESI-HR-MS (*m*/*z*): Calc. for C_17_H_20_N_2_PtS_2_Cl: 545.0389, 546.0410, 547.0411 Found: 545.0400, 546.0424, 547.0426. Elemental analysis, % calculated for C_17_H_20_N_2_PtS_2_: C, 39.91; H, 3.94; N, 5.48; Found: C, 39.58; H, 3.74; N, 5.32.

### PtIVDTh

To a solution of ligand **B** (0.046 g) in toluene (10 mL), complex [Pt_2_Me_4_(μ-SMe_2_)_2_] (0.055 g) was added, and the mixture was refluxed for 3 h. The solution was filtered through a 2 cm plug of Celite. The filtrate’s solvent was removed under vacuum and redissolved in 2 mL of DCM. A vial-in-vial recrystallization was performed using that solution and pentane. The resulting orangish solid was isolated and washed with 2 mL of ice cold ether and 3 × 2 mL of pentane and then dried under vacuum. Yield: 59 mg; 67%. ^1^H NMR (400 MHz, CDCl_3_) δ = 1.07 [s, 3H, ^2^*J*(PtH) = 75 Hz], 1.25 [s, 3H, ^2^*J*(PtH) = 73 Hz], 1.58 [m, 2H, Cy(H)], 1.88 [s, 3H, ^2^*J*(PtH) = 64 Hz], 2.03 [m, 6H, Cy(H)], 2.13 [s, 3H, ^2^*J*(PtH) = 68 Hz), 3.99 [m, 1H, Cy(H)], 5.67 [m, 1H, Cy(H)], 7.11 [m, 4H], 8.17 [s, 1H, ^3^*J*(PtH)=38 Hz], 8.49 (s, 1H, ^3^*J*_Pt–H_ = 46 Hz]. ^13^C NMR (100 MHz, CDCl_3_) δ = −7.6 (CH_3_) ^1^*J*(PtC) = 583 Hz, −1.0 (CH_3_) ^1^*J*(PtC) = 604 Hz, 1.9 (CH_3_) ^1^*J*(PtC) = 623 Hz, 2.3 (CH_3_) ^1^*J*(PtC) = 616 Hz, 25.4, 25.6, 35.0, 38.5, 63.2 *J*(PtC) = 11.0 Hz, 78.6, 124.8 *J*(PtC) = 38.0 Hz, 124.9 *J*(PtC) = 38.0 Hz, 125.0, 125.3 *J*(PtC) = 52.0 Hz, 141.7, 142.9, 149.9, 150.6, 163.3 ^2^*J*(PtC) = 44.0 Hz, 166.1 ^2^*J*(PtC) = 46.00 Hz. ESI-HR-MS (*m*/*z*): Calc. for C_20_H_28_Br_2_N_2_Pt_2_S_2_Cl: *m*/*z* 943.9003, 944.9024, 945.9026; found: 943.8952, 944.9014, 945.9074. Elemental analysis, % calculated for C_20_H_28_Br_2_N_2_Pt_2_S_2_: C, 26.38; H, 3.10; N, 3.08; Found: C, 27.22; H, 2.97; N, 2.98.

## Supporting Information Available

NMR spectra, IR spectra, emission spectral data, orbital plots, and PDF data (PDF)
Compound **PtIIMTh** (CIF)
Compound **PtIVDTh** (CIF)

## Supplementary Material

ao3c05567_si_001.pdf

ao3c05567_si_002.cif

ao3c05567_si_003.cif
