# Supplementary material for: Synthesis, Structure, and Photophysical Properties of Platinum Compounds with Thiophene-Derived Cyclohexyl Diimine Ligands
Source: ACS Omega. 2023 Oct 6;8(41):38587–96. doi: 10.1021/acsomega.3c05567 (PMC10586441; doi:10.1021/acsomega.3c05567)
Supplement: Supplementary file 1 — ao3c05567_si_001.pdf [file ao3c05567_si_001.pdf]

Supporting Information

Synthesis, Structure, and Photophysical Properties of Platinum Compounds with Thiophene-Derived Cyclohexyl Diimine Ligands

Matthew W. Greenberg,[a] Kris M. Tulloch, Michelle E. Reynoso,[a] Juliette L. Knapp[a], Farman H. Sayem,[a] Daphne D. Bartkus,[a] Ryan Lum, Christopher N. LaFratta,[a] Joseph M. Tanski,[b] Craig M. Anderson,*[a]

[a] Department of Chemistry & Biochemistry, Bard College, 30 Campus Road, Annandale-on-Hudson, NY, 12504, USA.

Email: canderso@bard.edu Phone: 845-752-2356. FAX: 845-752-2339

[b] Department of Chemistry, Vassar College, Poughkeepsie, NY, 12604, USA.

Table of Contents

NMR Spectra	2-9
IR Spectra	10-12
Photophysical Data: UV/Vis, Emission Spectra, Lifetime Data	13-19
Exponetial Decay Curves	20-25
DFT Orbitals	26-33
TD-DFT Results	34-35
X-ray Pair Distribution Function Analysis Data	36-39

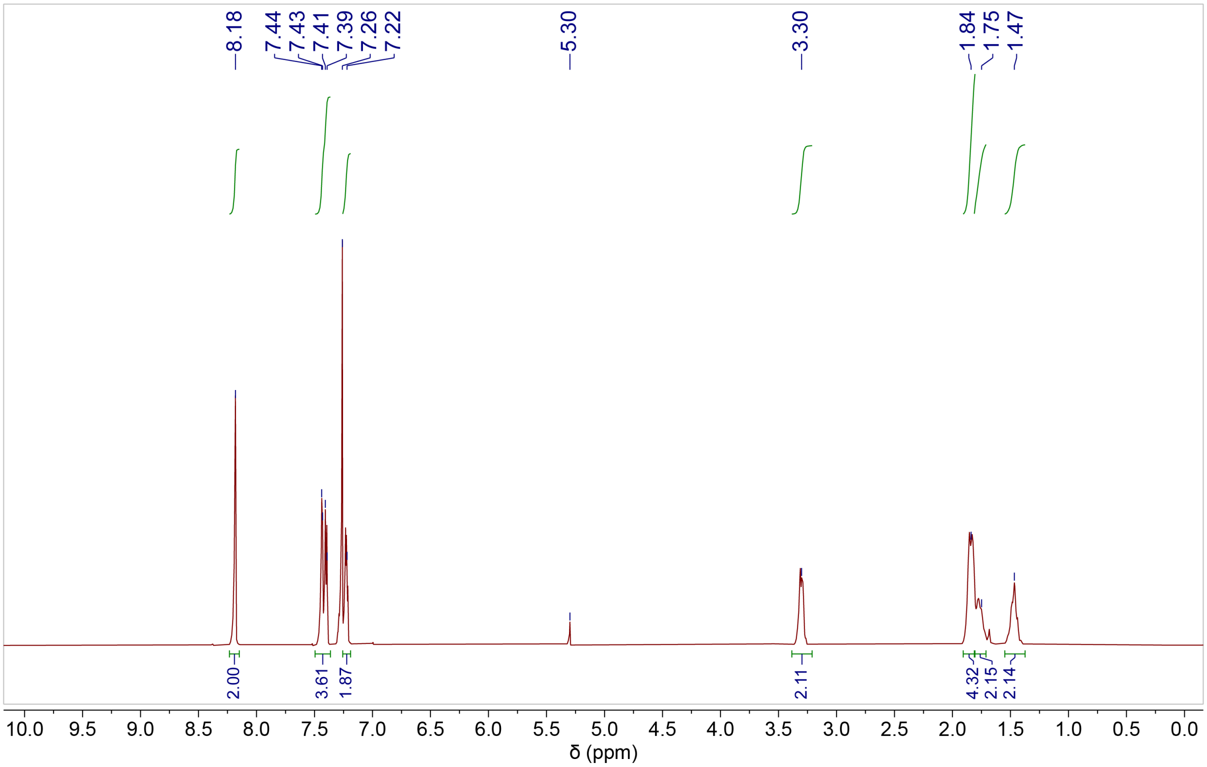

Figure S1. ^{1}H NMR of **Ligand A**

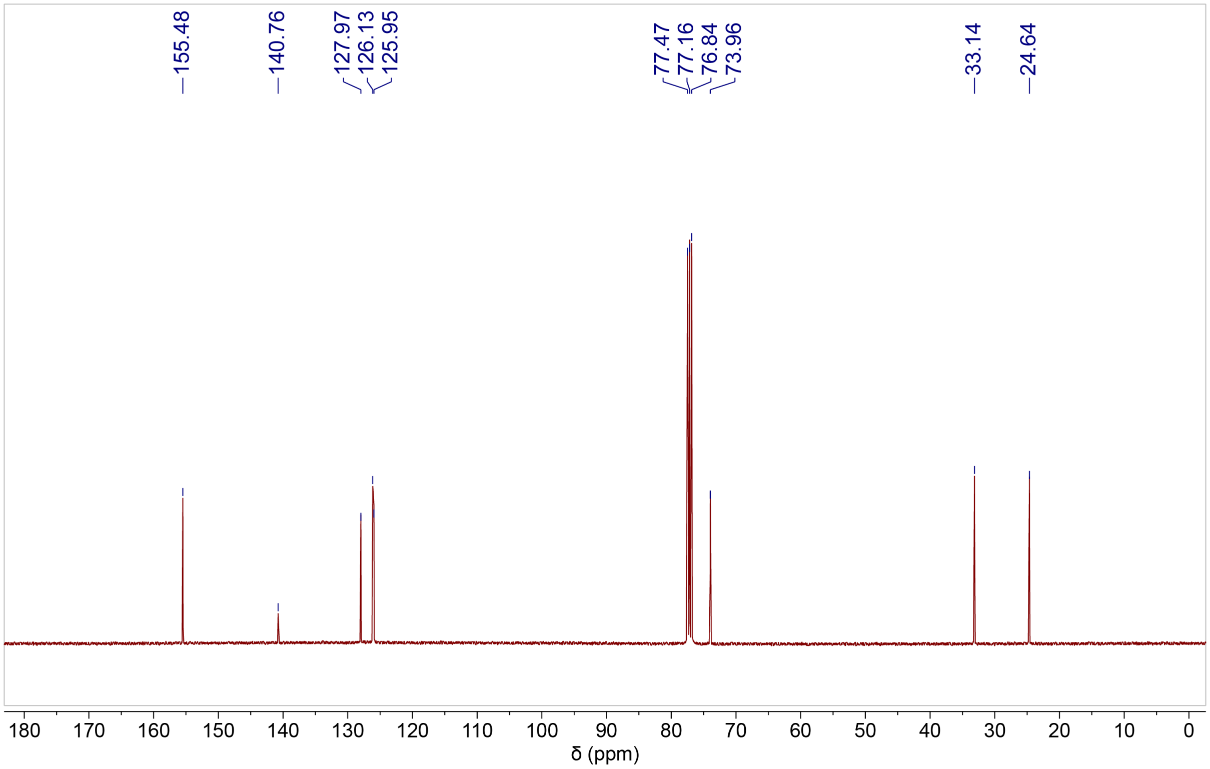

Figure S2. ^{13}C NMR of **Ligand A**

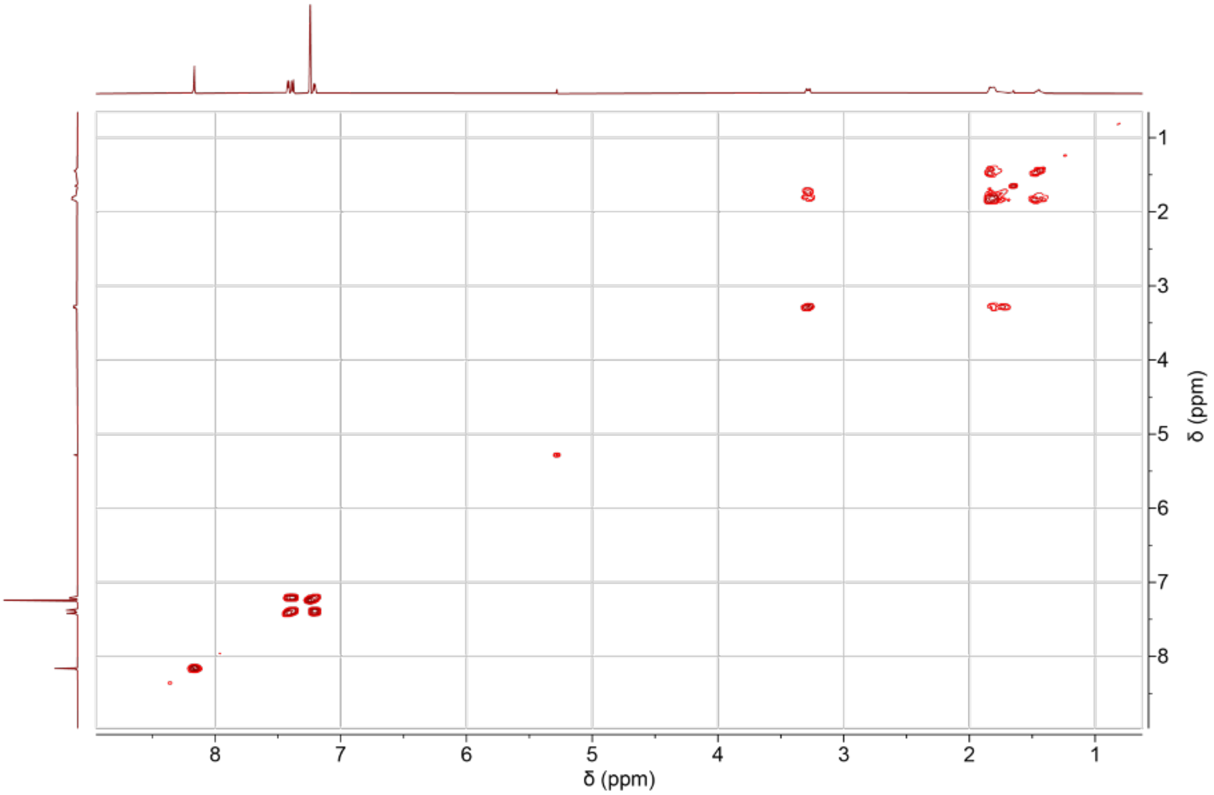

Figure S3. ^{1}H-^{1}H gCOSY NMR of **Ligand A**

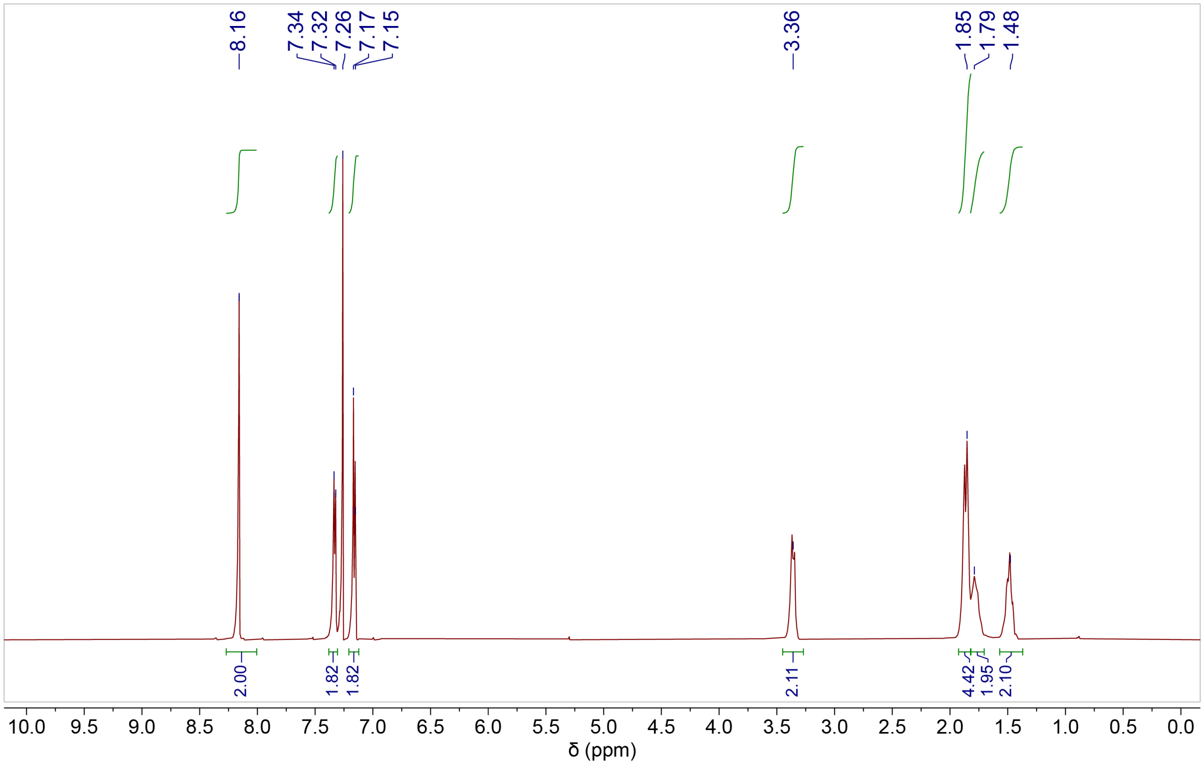

Figure S4. ^{1}H NMR of **Ligand B**

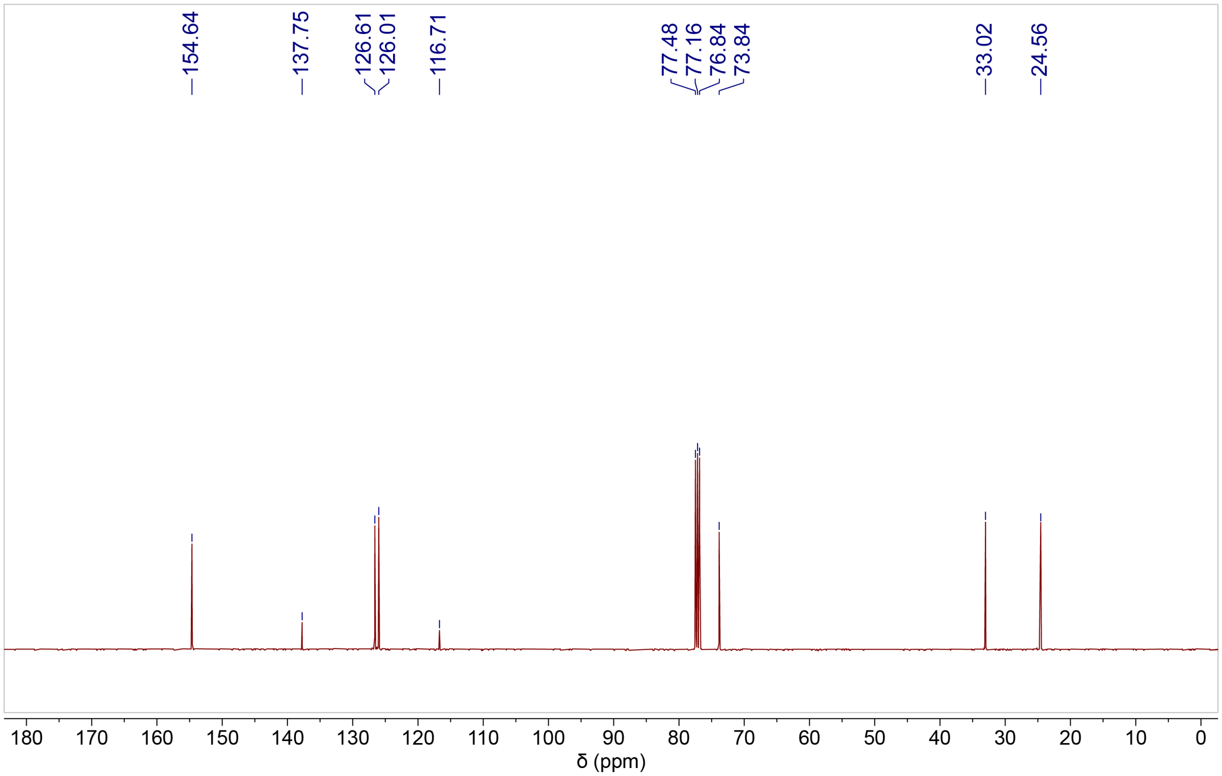

Figure S5. ^{13}C NMR of **Ligand B**

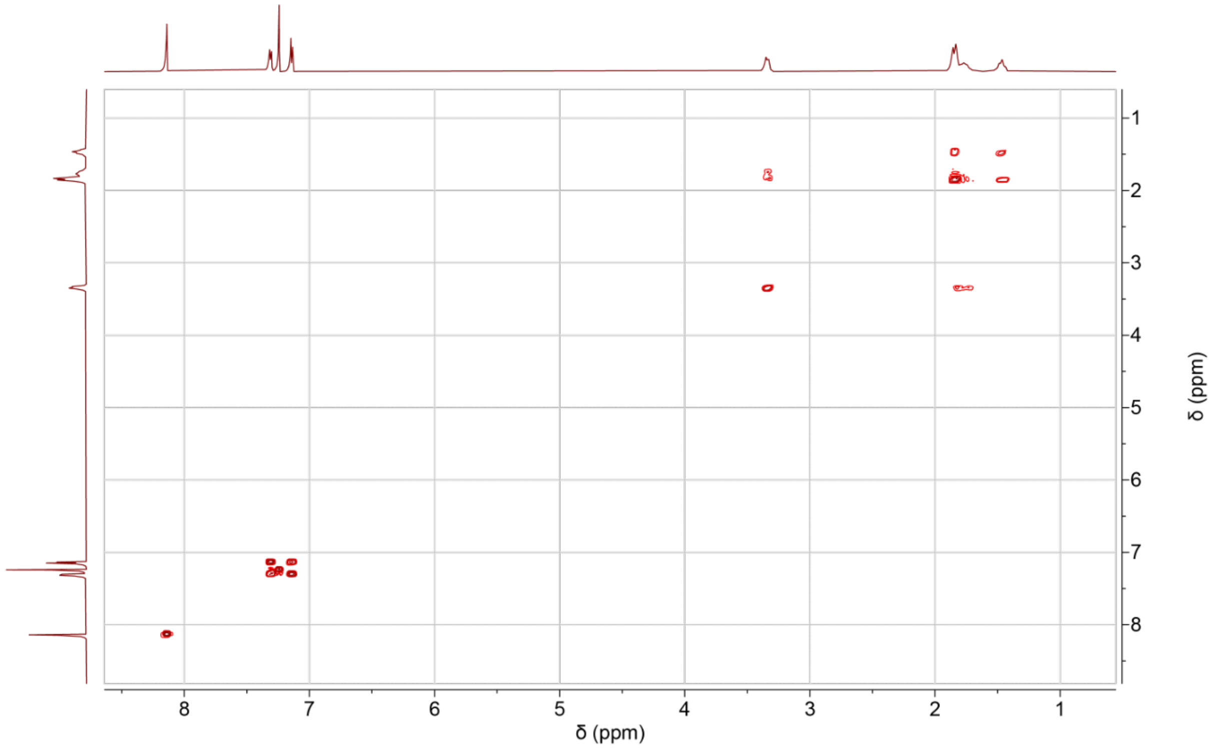

Figure S6. ^{1}H-^{1}H gCOSY NMR of **Ligand B**

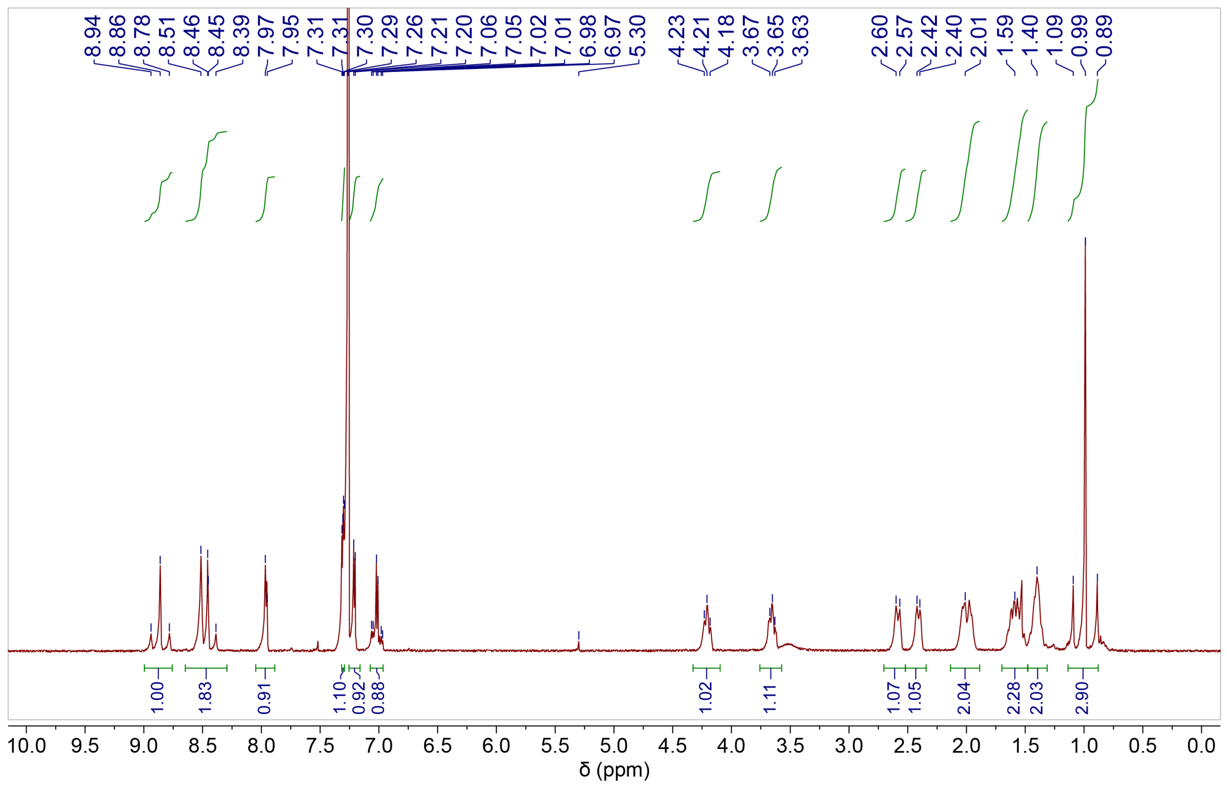

Figure S7. ^{1}H NMR of **PtIIMTh**

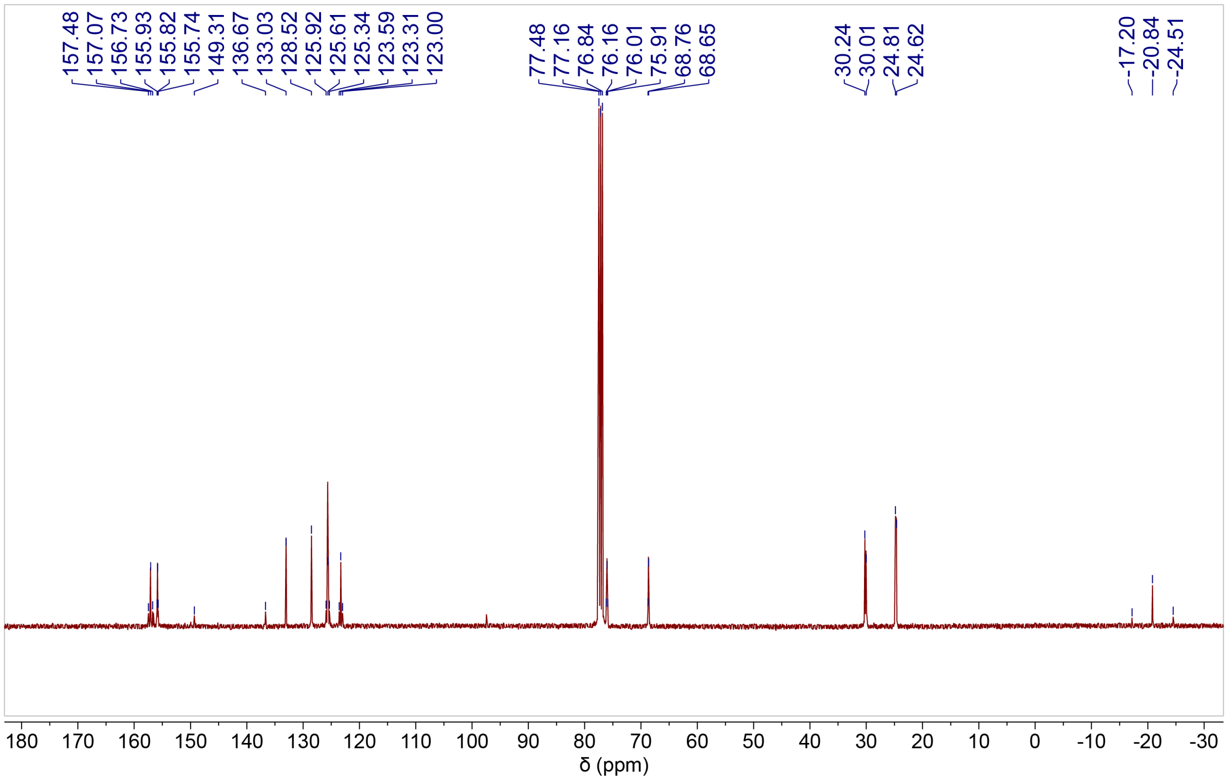

Figure S8. ^{13}C NMR of **PtIIMTh**

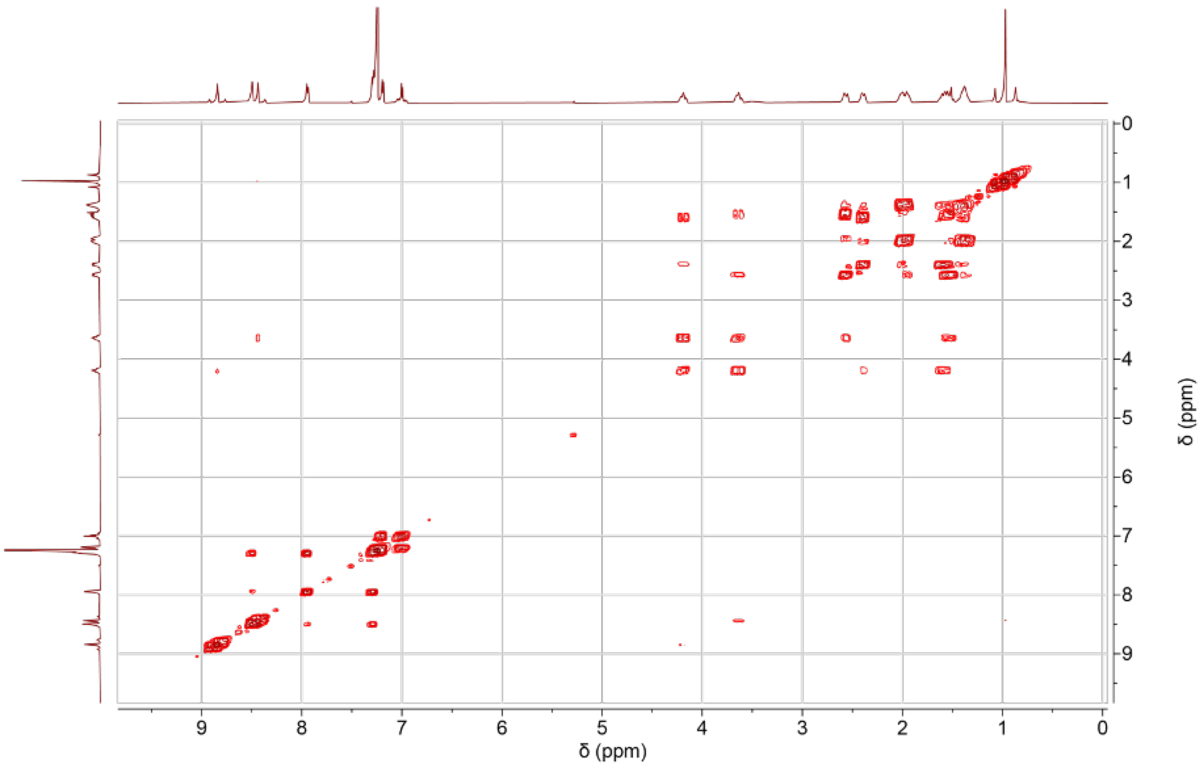

Figure S9. ^{1}H-^{1}H gCOSY NMR of **PtIIMTh**

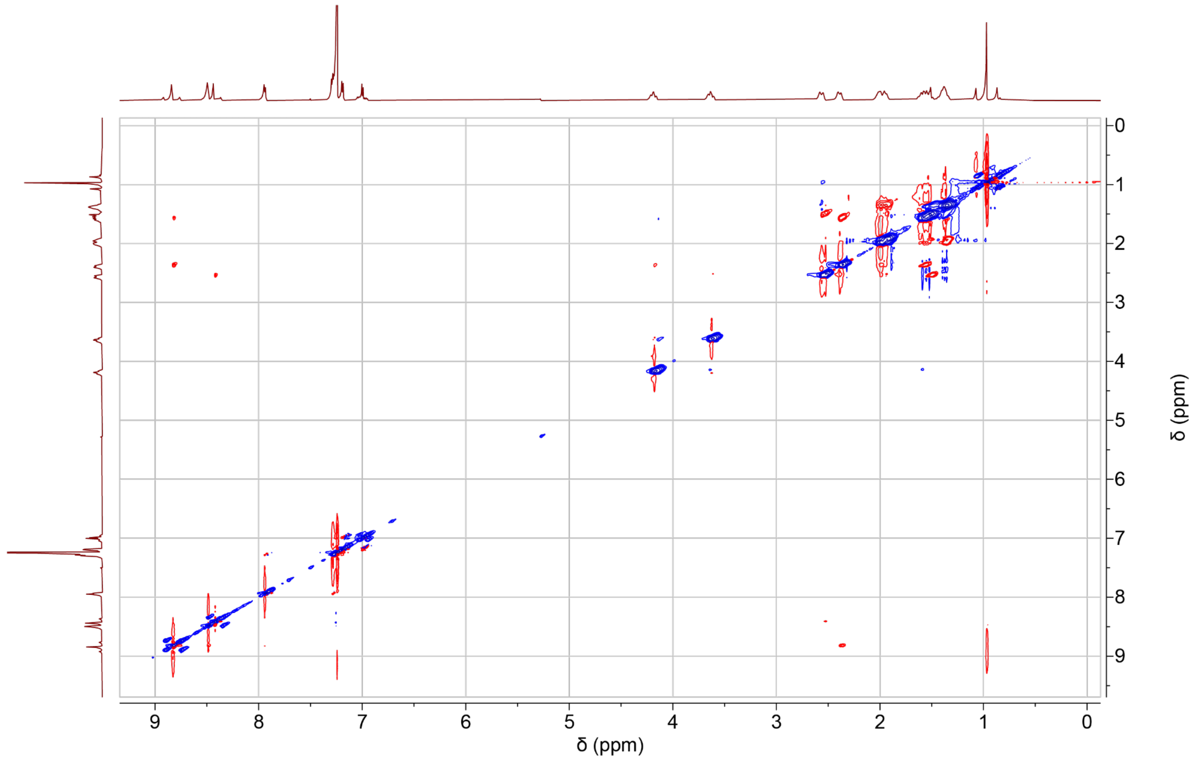

Figure S10. ^{1}H-^{1}H NOESY NMR **PtIIMTh**

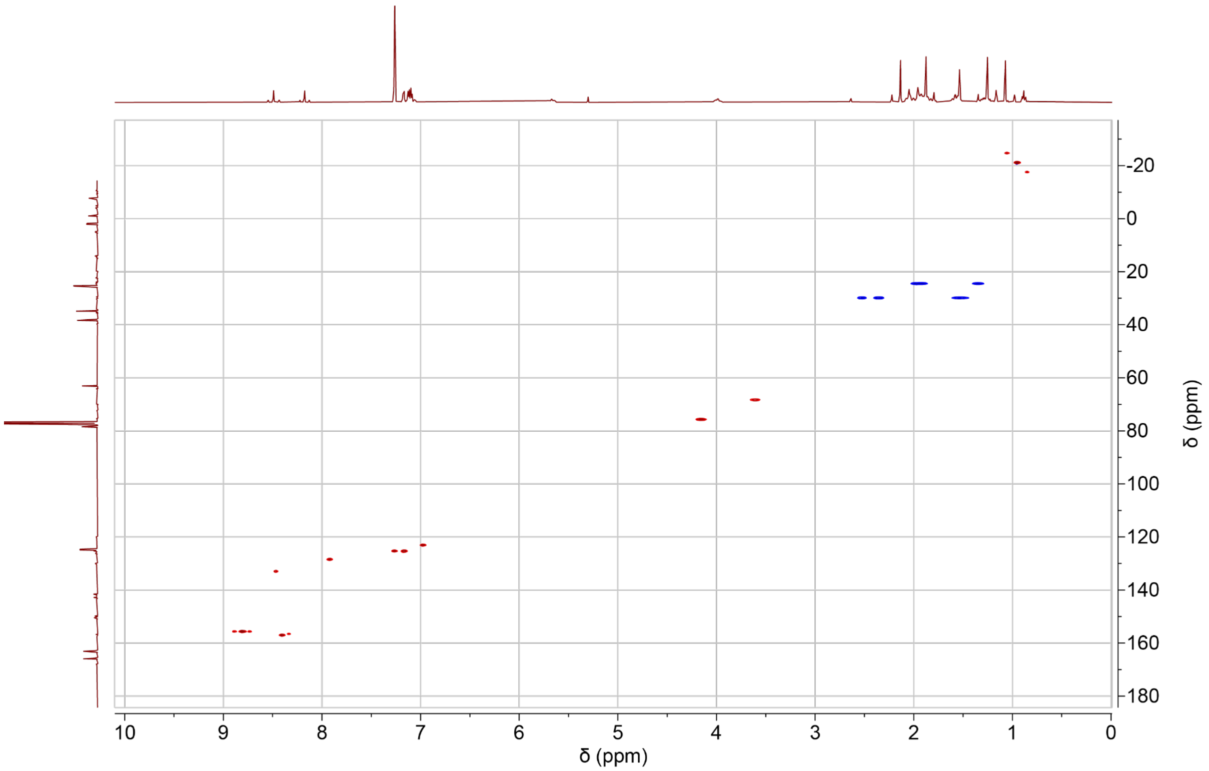

Figure S11. ^{1}H-^{13}C HSQCAD NMR **PtIIMTh**

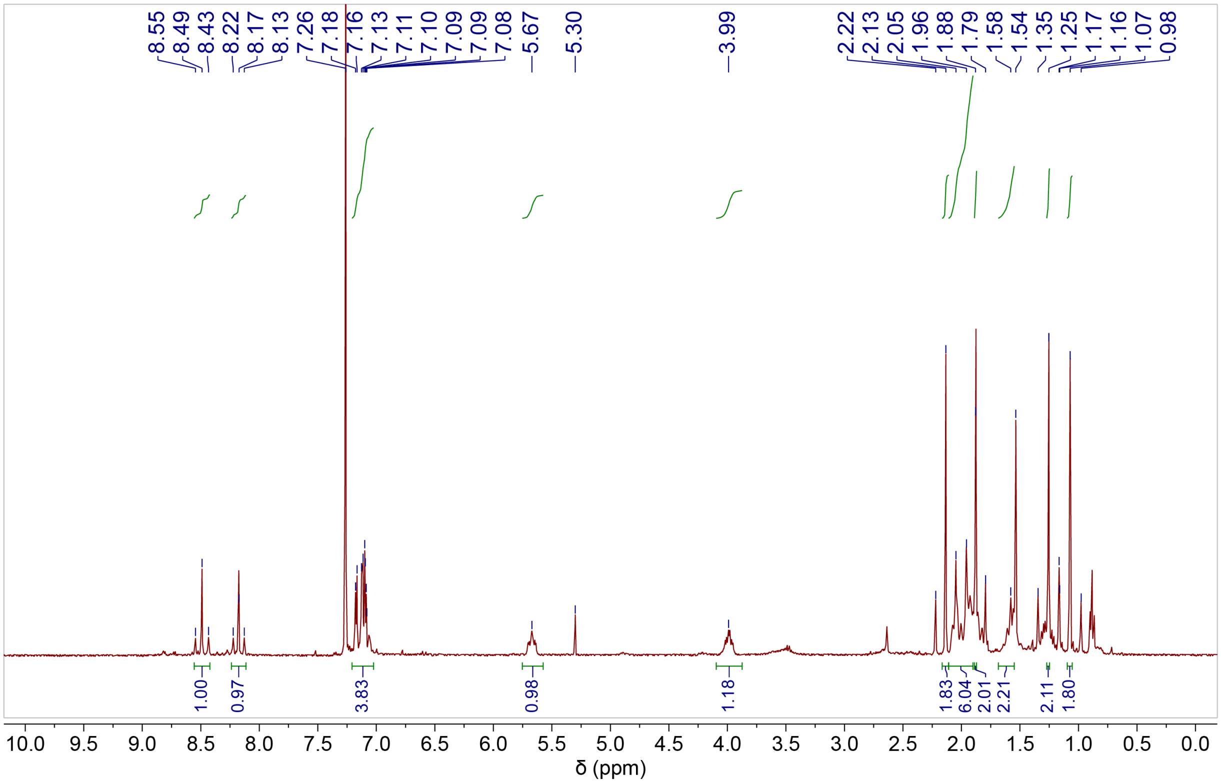

Figure S12. ^{1}H NMR of **PtIVDTh**. For the four Pt-Me resonances, the central peak excluding the two ^{195}Pt satellites are integrated due to overlapping chemical shifts in this region of the spectrum.

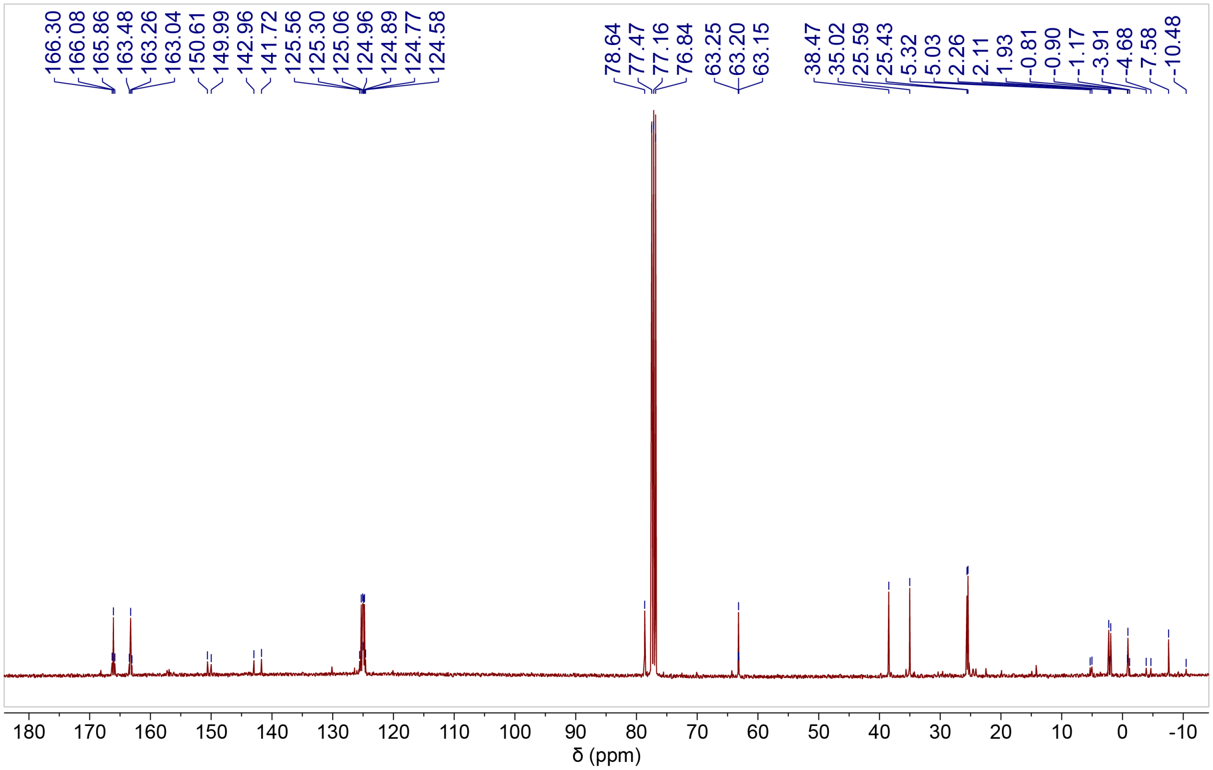

Figure S13. ^{13}C NMR of **PtIVDTh**

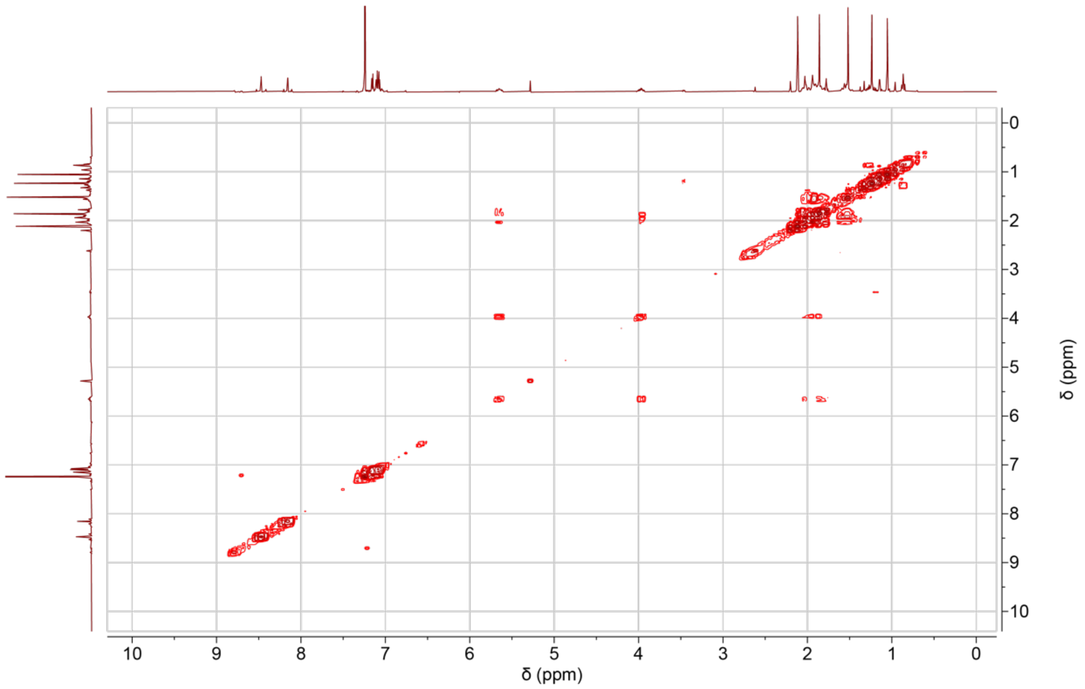

Figure S14. ^{1}H-^{1}H gCOSY NMR **PtIVDTh**

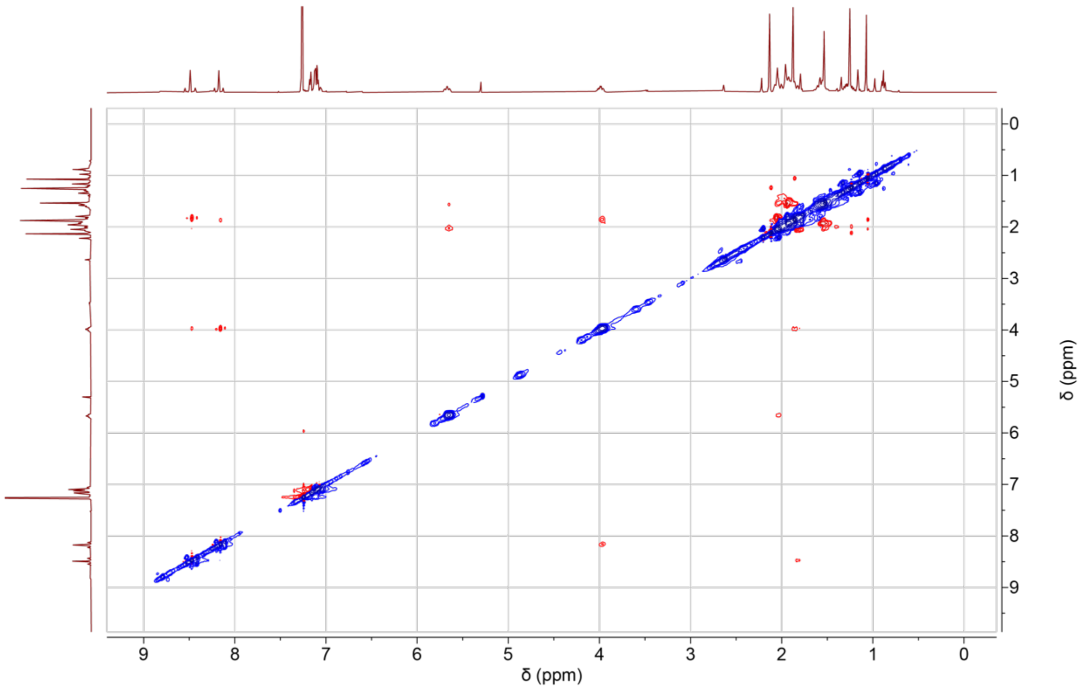

Figure S15. ^{1}H-^{1}H NOESY NMR **PtIVDTh**

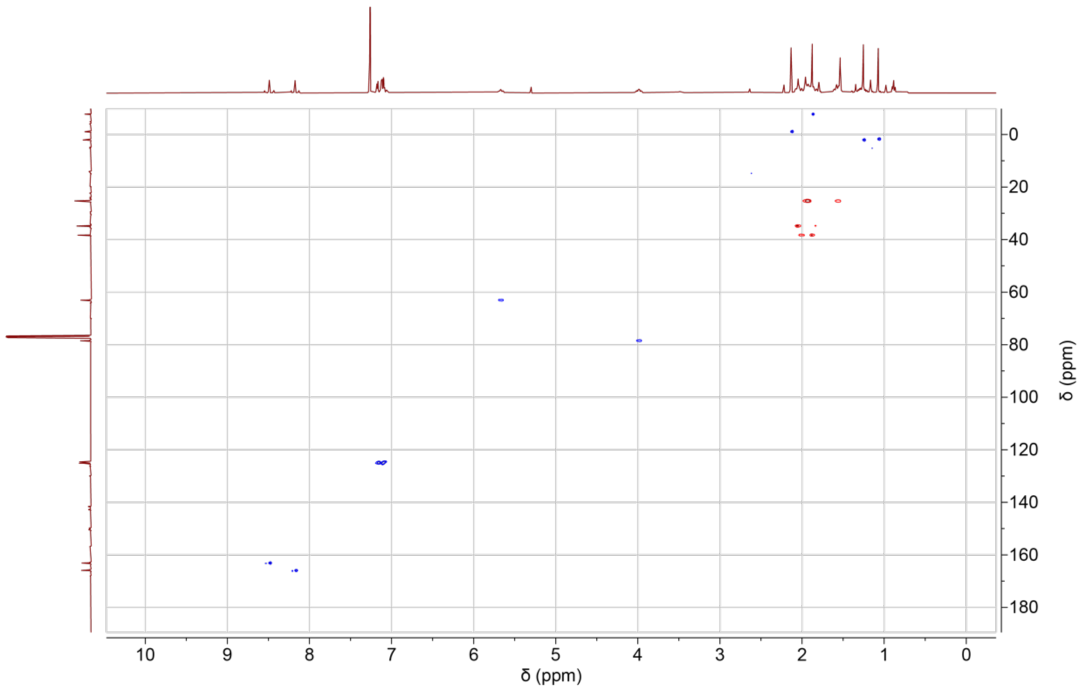

Figure S16. ^{1}H-^{13}C HSQCAD NMR **PtIVDTh**

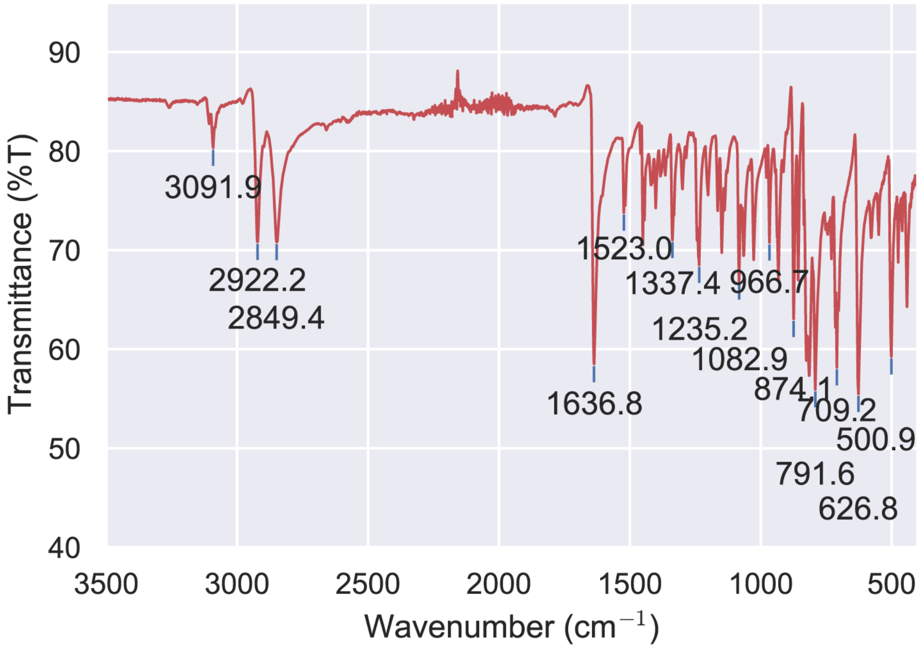

Figure S17. Transmittance FT-IR of **Ligand A**

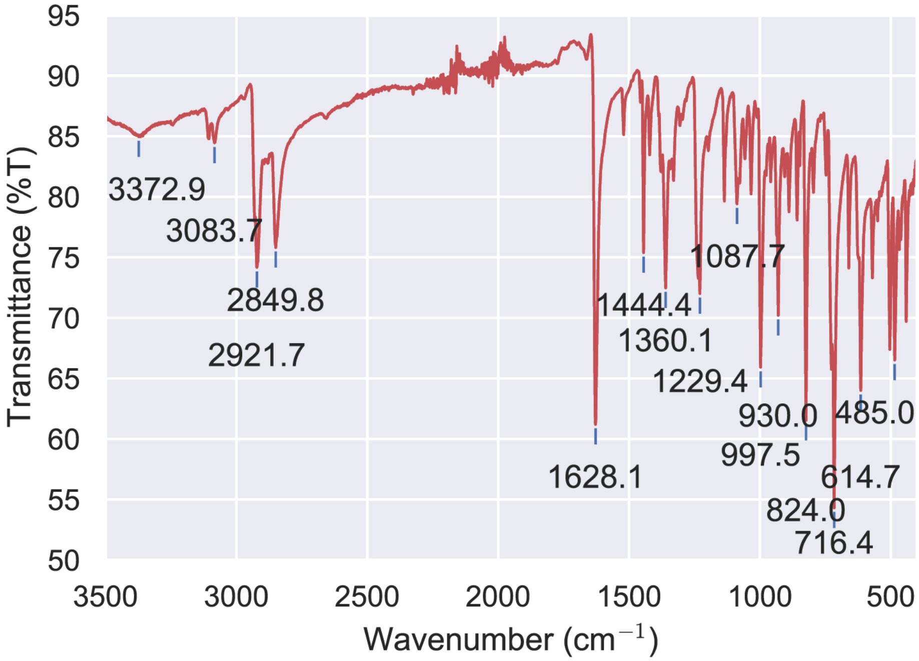

Figure S18. Transmittance FT-IR of **Ligand B**

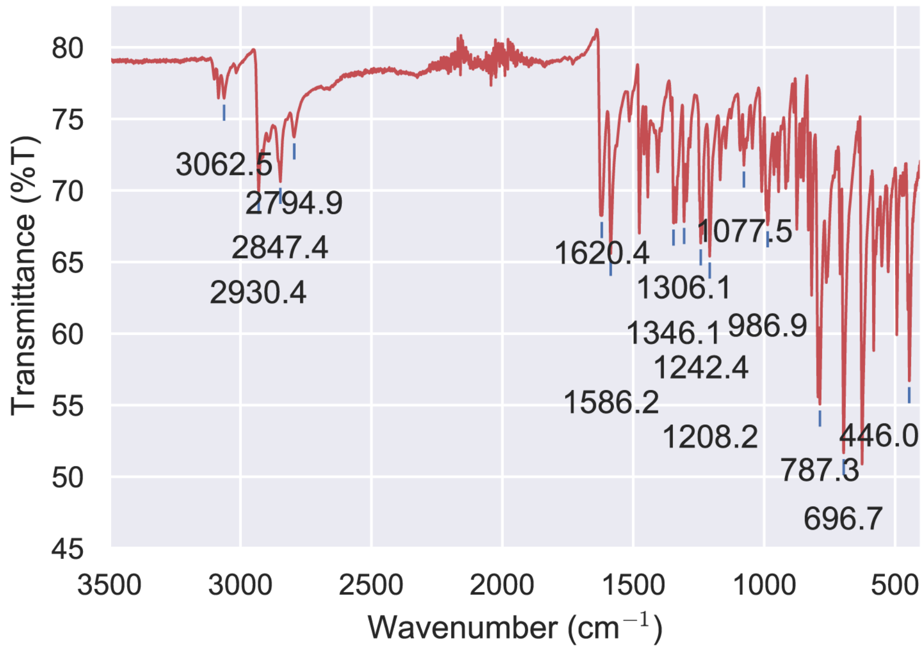

Figure S19. Transmittance FT-IR of **PtIIMTh**

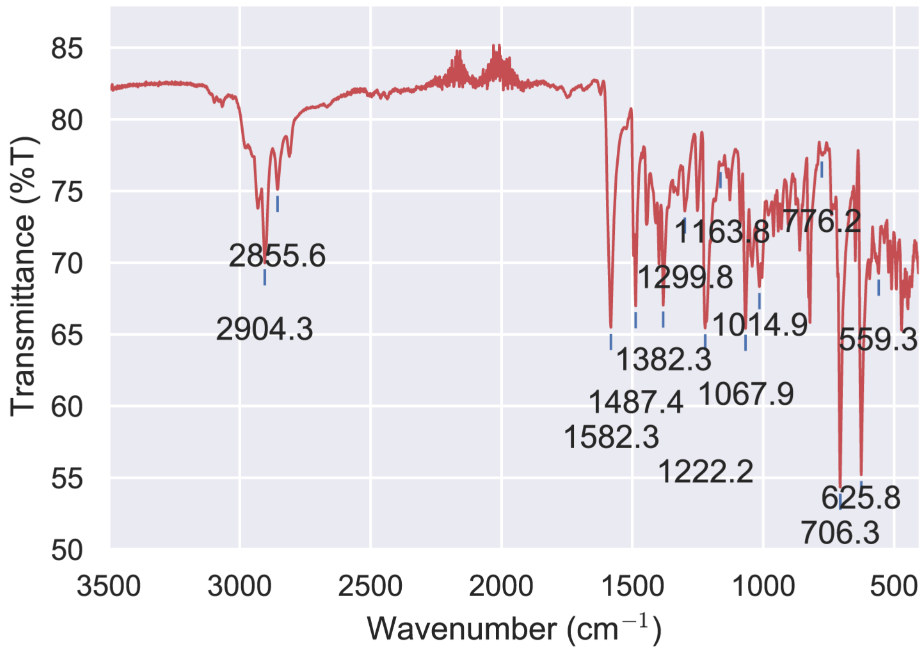

Figure S20. Transmittance FT-IR of **PtIVDTh**

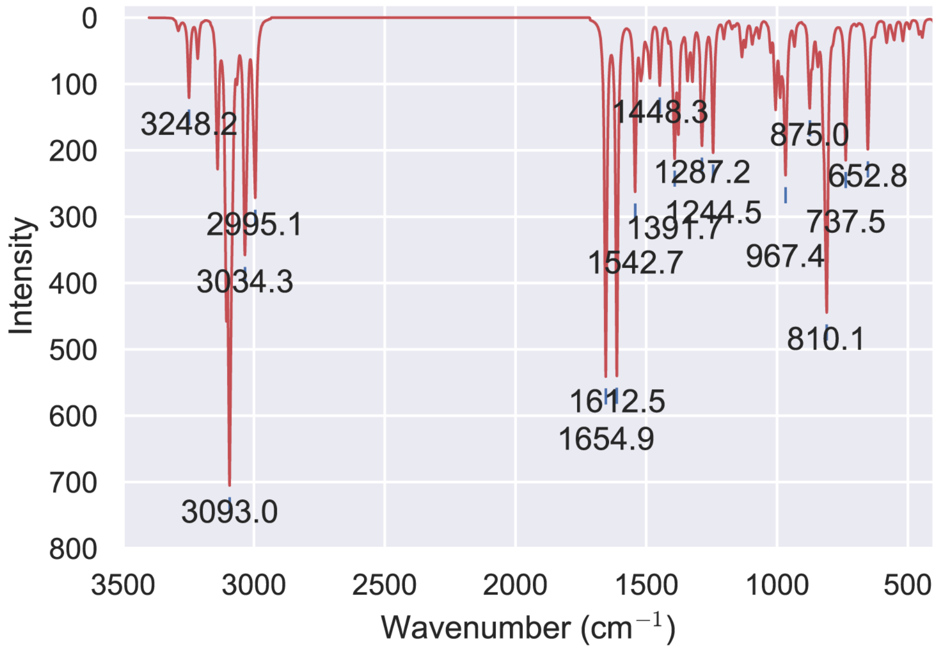

Figure S21: DFT calculated IR spectrum of **PtIIMTh** (10 nm linewidth)

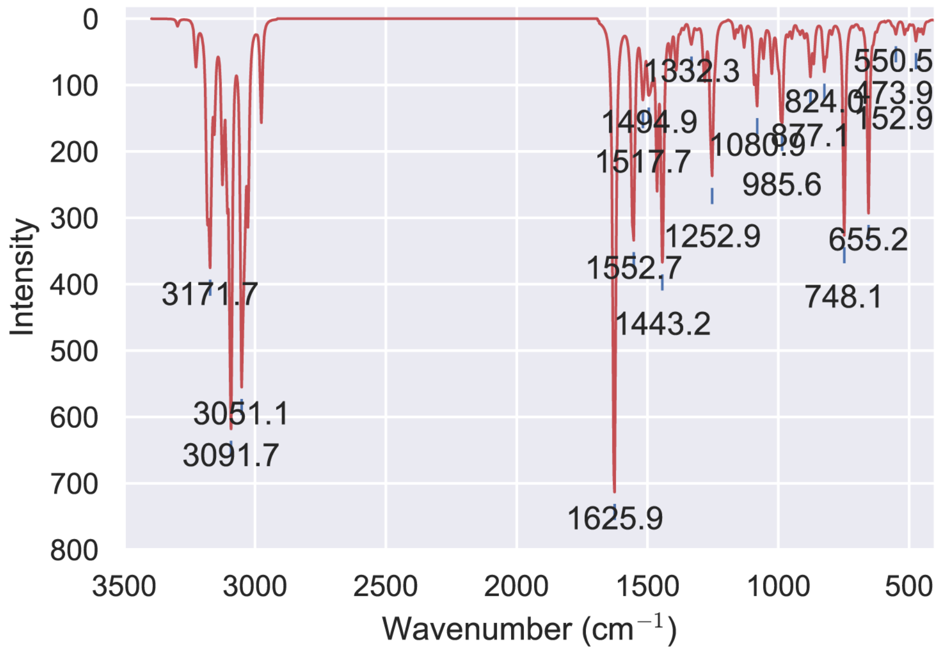

Figure S22: DFT calculated IR spectrum of **PtIVDTh (**10 nm linewidth).

Table S1: UV/vis Absorbance Data

ABSORPTION		
Compound	Absorption peak (nm)	Extinction coefficient (M-1 cm-1)
**Pt(II)Mth**	325	6400
	350	5900
	393	4200
	450	1800
**Pt(IV)Dth**	350	3700
	280	12300
**Pt(II)MPh**	340	8000
	415	4800
**Pt(IV)DPh**	335	4000

Table S2: Emission Spectroscopy Data

EMISSION (SOLUTION STATE)		
Compound	Excitation (nm)	Emission in solution state (nm)
**Pt(II)Mth**	350	580
		620
		670
	450	595
		635
		680
**Pt(IV)Dth**	350	430
		550
**Pt(II)MPh**	350	470
		503
		543
	400	545
	450	595
		621
**Pt(IV)DPh**	350	483
		511
		550

EMISSION (SOLID STATE)		
Compound	Excitation (nm)	Emission in solid state (nm)
**Pt(II)Mth**	400	613
		665
		723
	450	565
		605
		685
	500	505
		552
		618
**Pt(IV)Dth**	400	685
		735
	450	630
**Pt(II)MPh**	350	625
	400	625
**Pt(IV)DPh**	400	570

Table S3: Excited State Lifetime Data

LIFETIME in SOLUTION				
Compound	Excitation LED (nm)	Emission in solution (nm)	Lifetimes in solution (ns)	chi^2
**Pt(II)Mth**	450	585	215.8	1.019
	450	610	210.9	1.089
	450	670	190.2	1.059
**Pt(IV)Dth**	365	440	37.4	1.154
	365	550	92.4	1.101
**Pt(II)MPh**	450	615	103.2	0.9747
**Pt(IV)DPh**	365	480	137.2	1.252
	365	510	161.8	1.136
	365	550	175.8	1.132

LIFETIME in SOLID STATE				
Compound	Excitation LED (nm)	Emission in solid state (nm)	Lifetimes in solid state (ns)	Chi ^2
**Pt(II)Mth**	450	610	264	1.09
	450	645	269	1.07
	450	725	343	0.95
**Pt(IV)Dth**	450	675	213	1.15
	450	725	209	1
**Pt(II)MPh**	405	620	126	1.22
	405	675	134	1.28
**Pt(IV)DPh**	365	575	193	1.04
	405	575	180	1.16
	405	625	170	1.13
	405	715	168	1.18

LIFETIME in PMMA FILMS				
Compound	Excitation LED (nm)	Emission in solid state (nm)	Lifetimes in solid state (ns)	Chi ^2
**Pt(II)Mth**	405	650	23, 167	1.16
**Pt(IV)Dth**	405	615	24, 435	1.02
**Pt(II)MPh**	405	610	13.6, 207	1.17
**Pt(IV)DPh**	405	550	12, 236	1.12

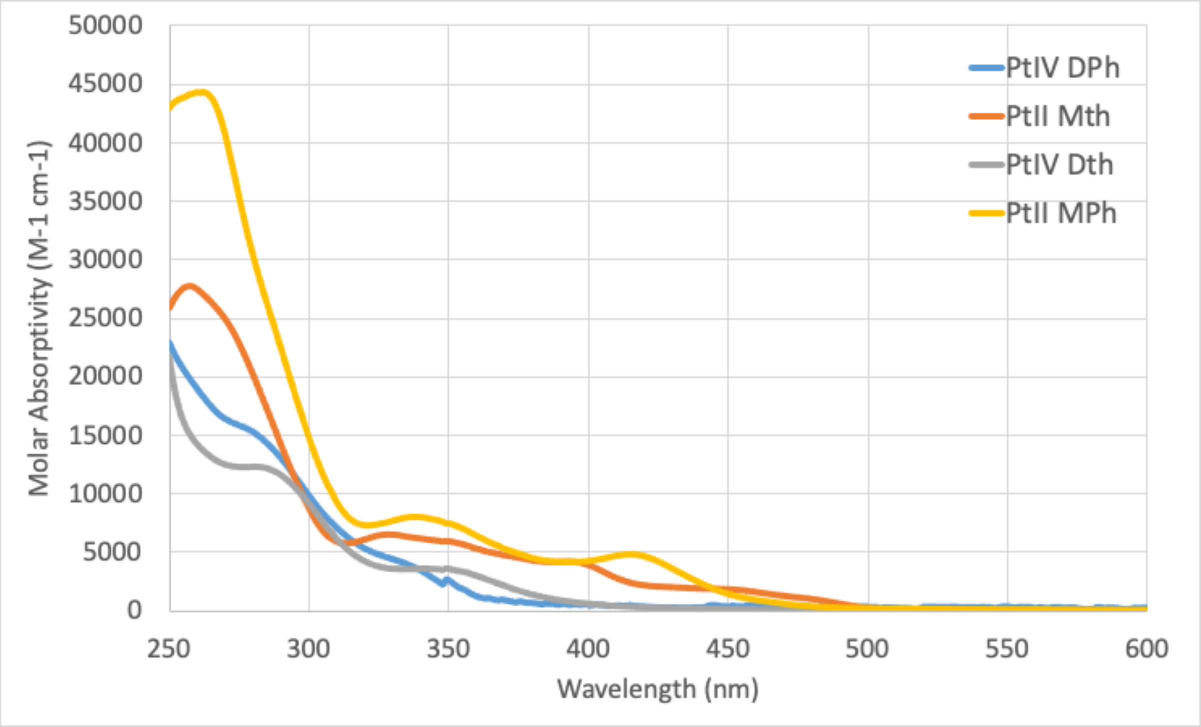

Figure S23: UV-vis absorption spectra in DCM

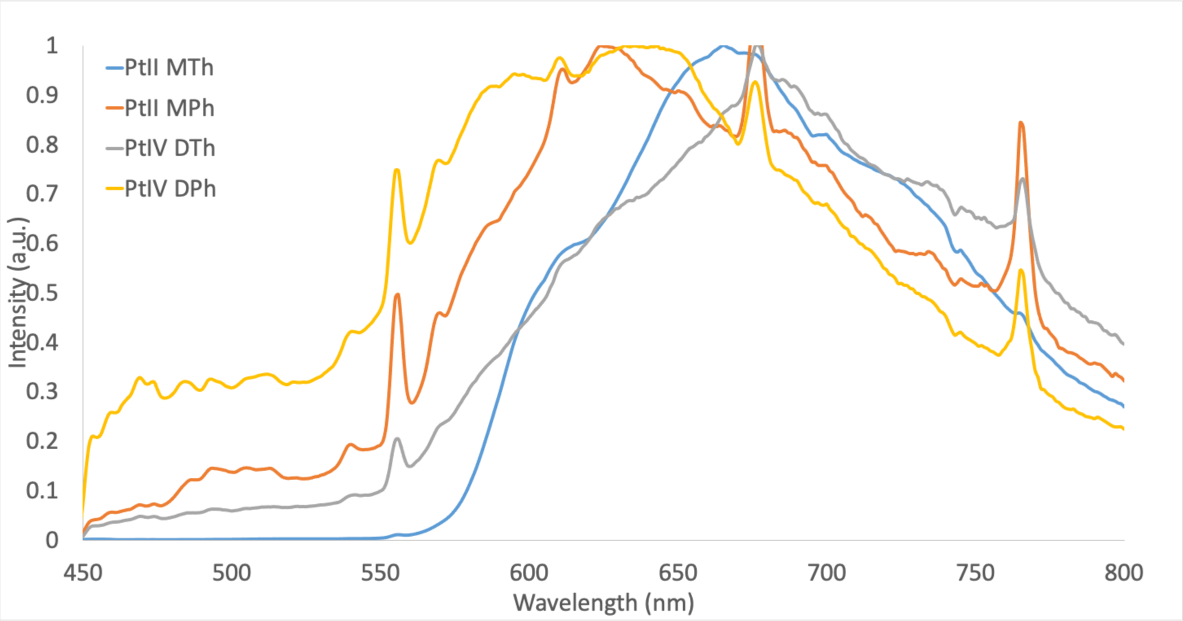

Figure S24: Solid State Emission spectra excited at 400 nm

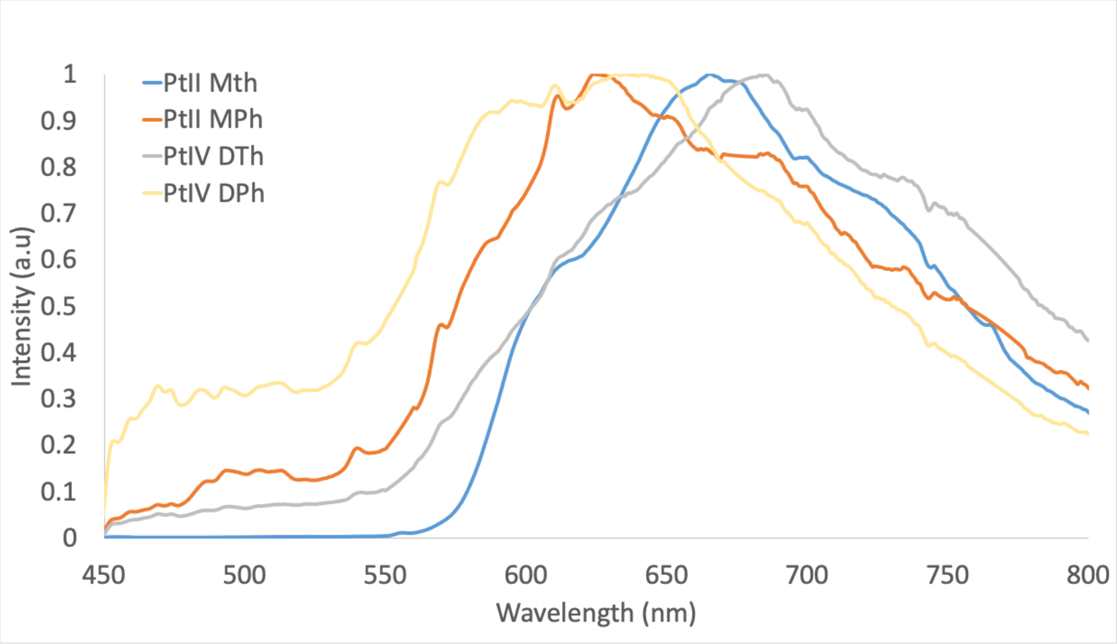

Figure S25: Solid State Emission spectra excited at 400 nm with Raman subtracted

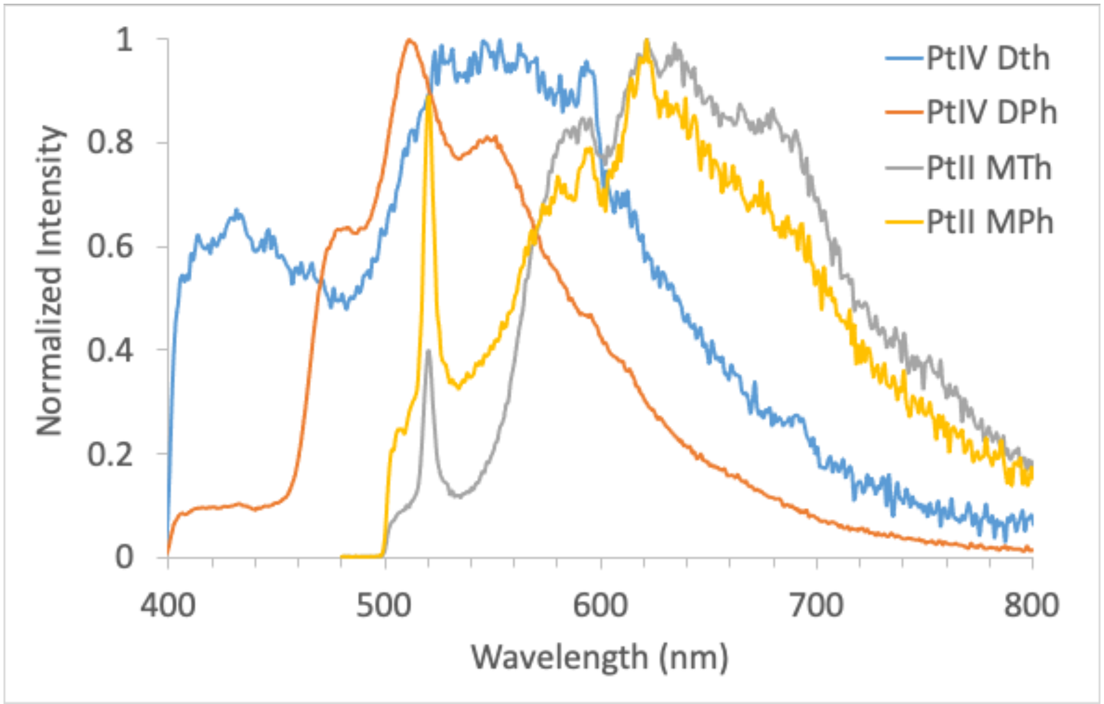

Figure S26: Steady state emission spectra in DCM, Pt(IV) compound excited at 350 nm and Pt(II) compounds excited at 450 nm.

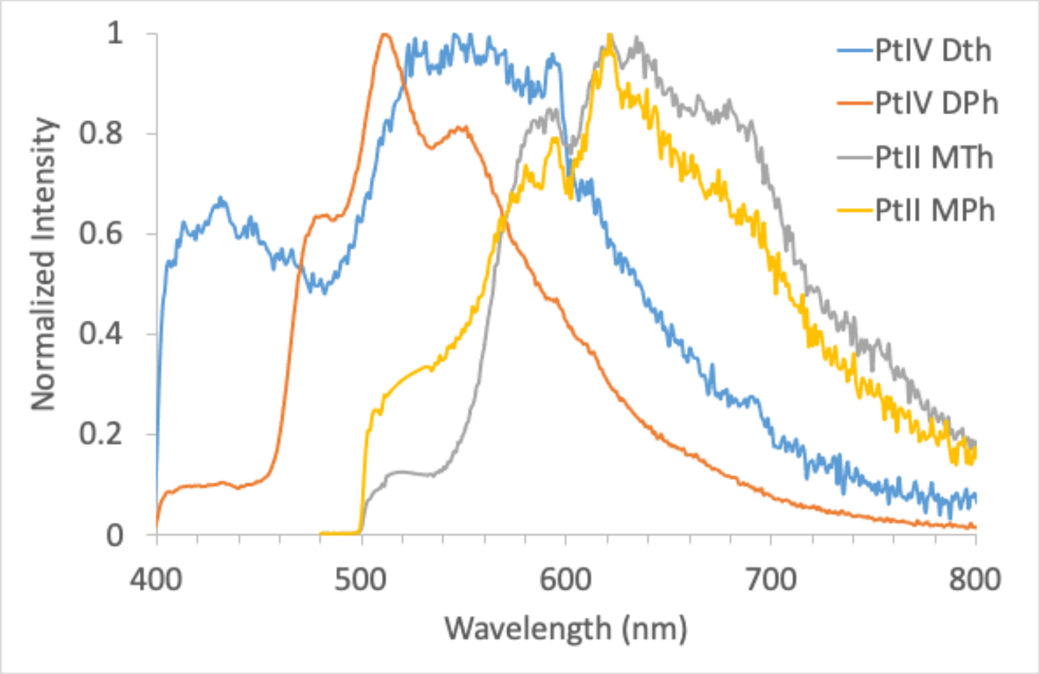

Figure S27: Steady state emission spectra, Pt(IV) compounds excited at 350 nm and Pt(II) compounds excited at 450 nm, Raman removed.

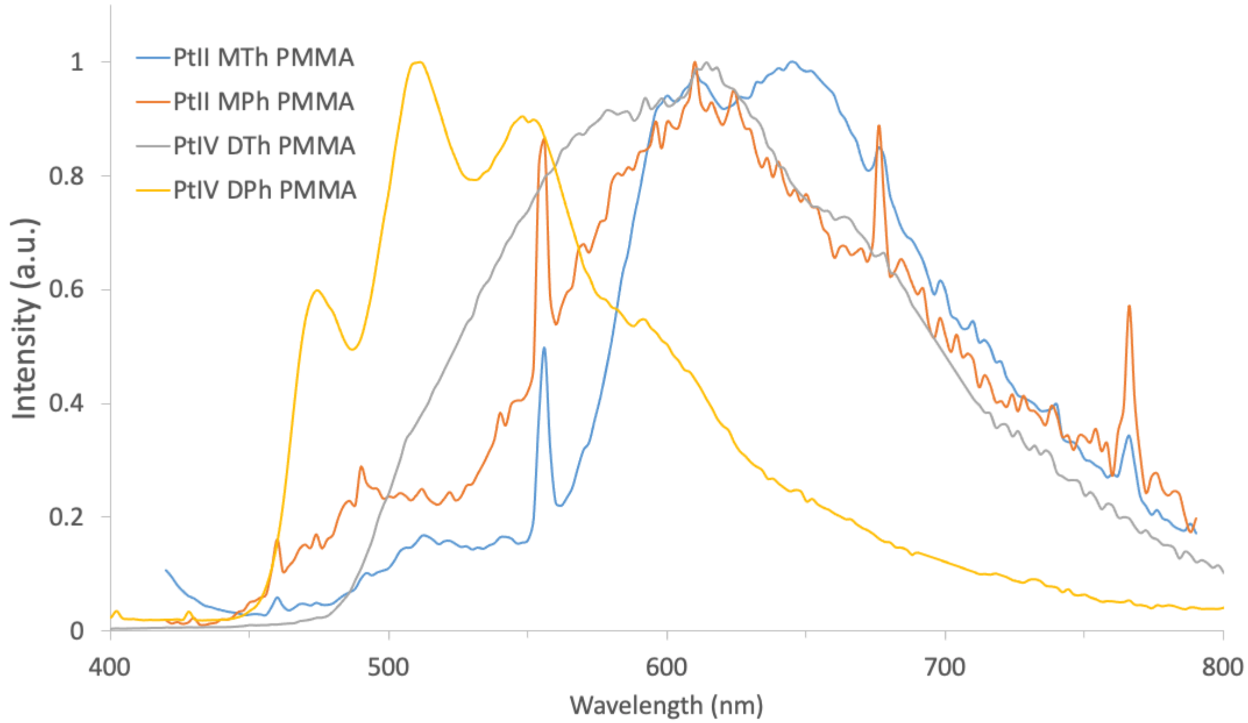

Figure S28: Steady state emission spectra in PMMA doped films. Pt(IV) compounds excited at 350 nm and Pt(II) compounds excited at 400 nm.

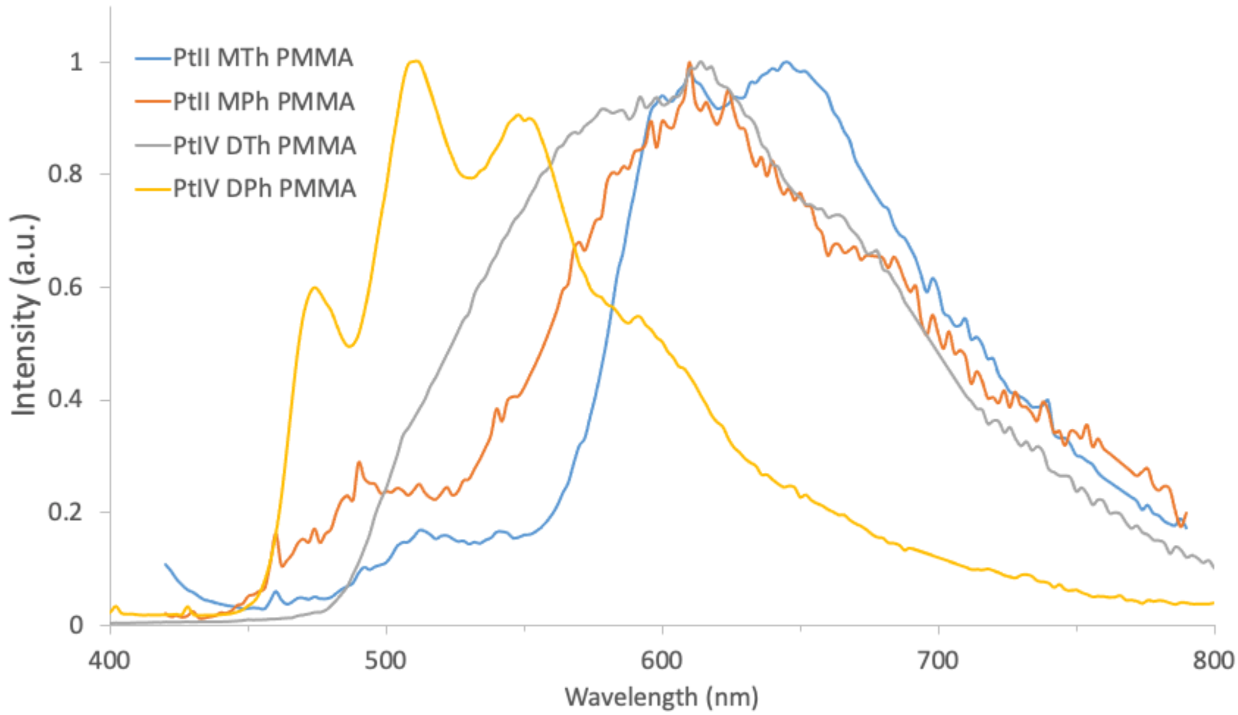

Figure S29: Steady state emission spectra in PMMA doped films. Pt(IV) compounds excited at 350 nm and Pt(II) compounds excited at 400 nm, Raman removed.

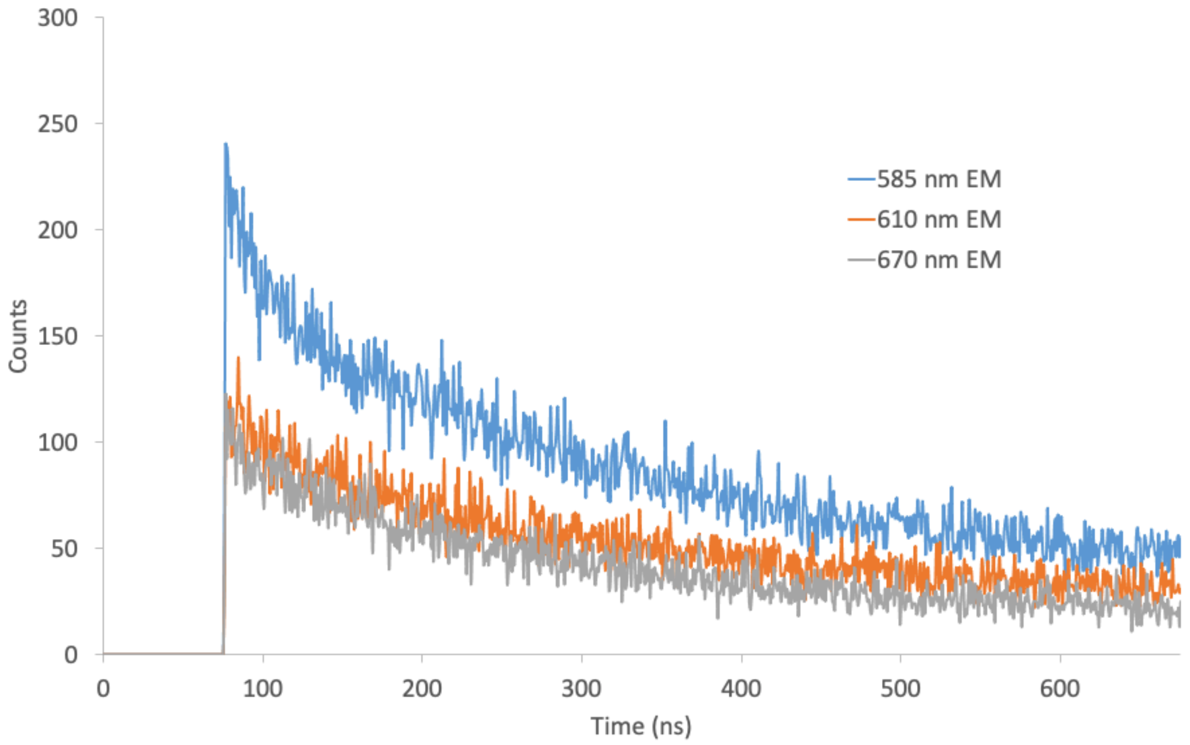

Figure S30. TCSPC decay curve for Pt(II)MTh in the DCM excited at 450 nm.

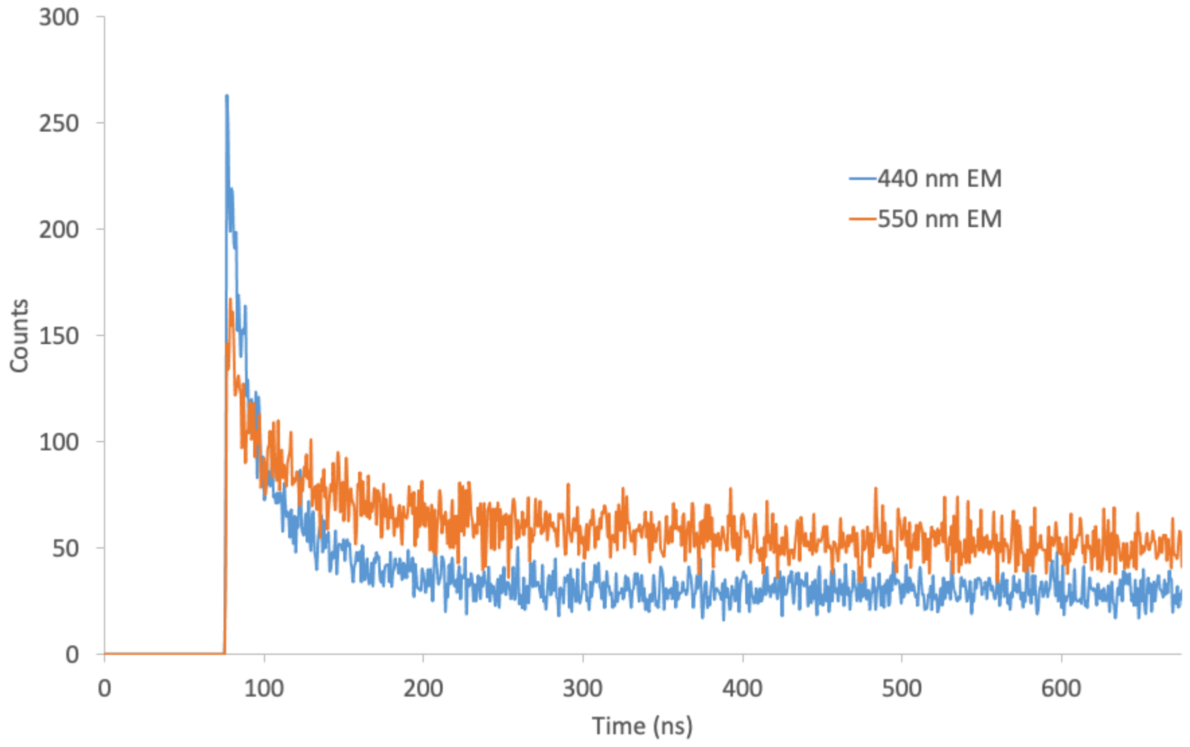

Figure S31. TCSPC decay curve for Pt(IV)DTh in the DCM excited at 365 nm.

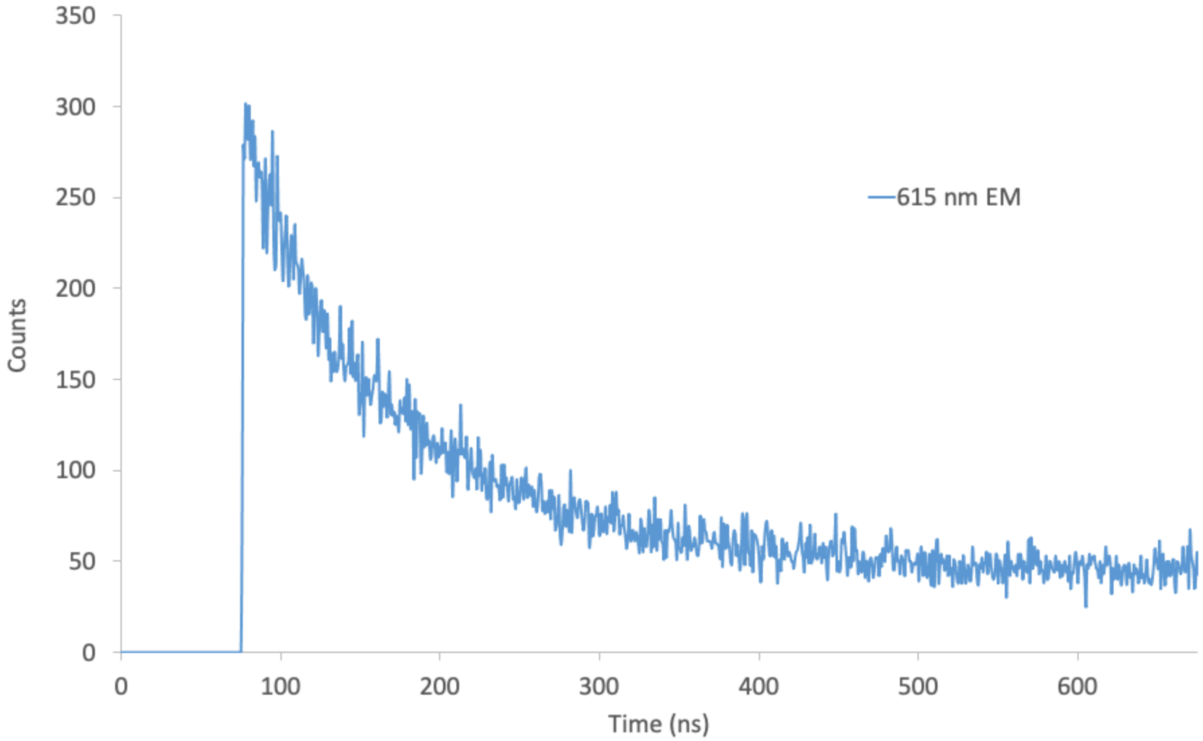

Figure S32. TCSPC decay curve for Pt(II)MPh in the DCM excited at 450 nm.

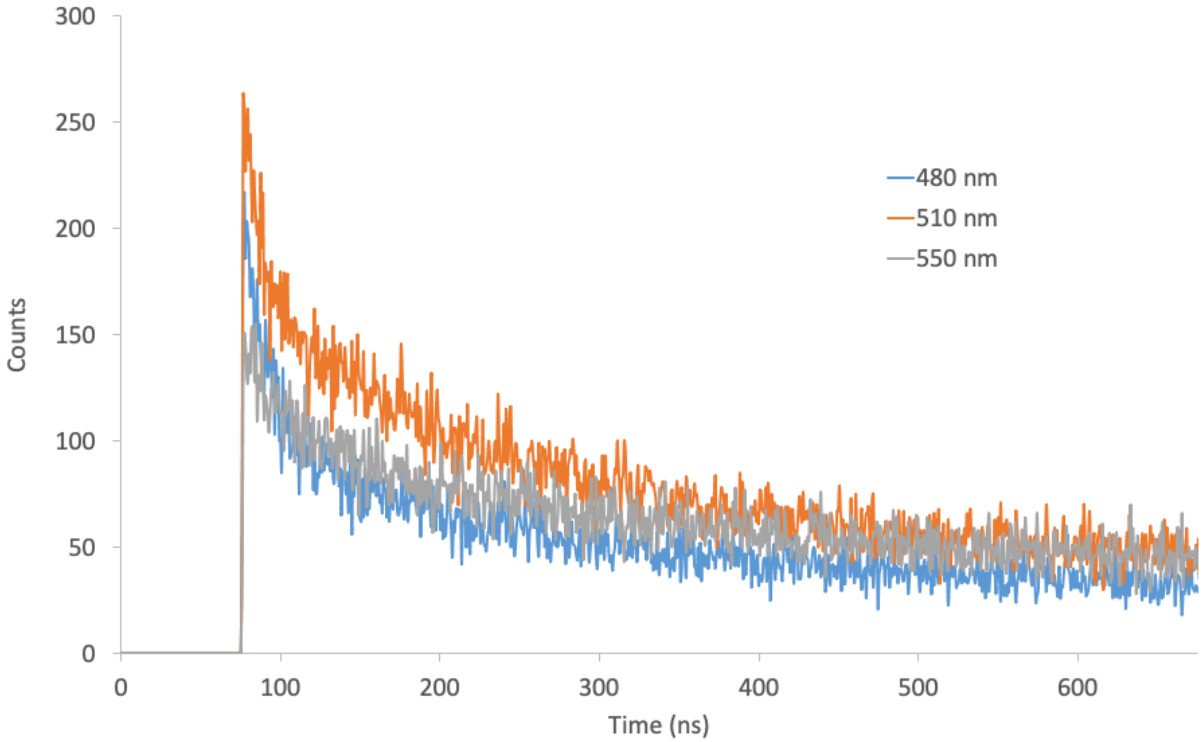

Figure S33. TCSPC decay curve for Pt(IV)DPh in the DCM excited at 365 nm.

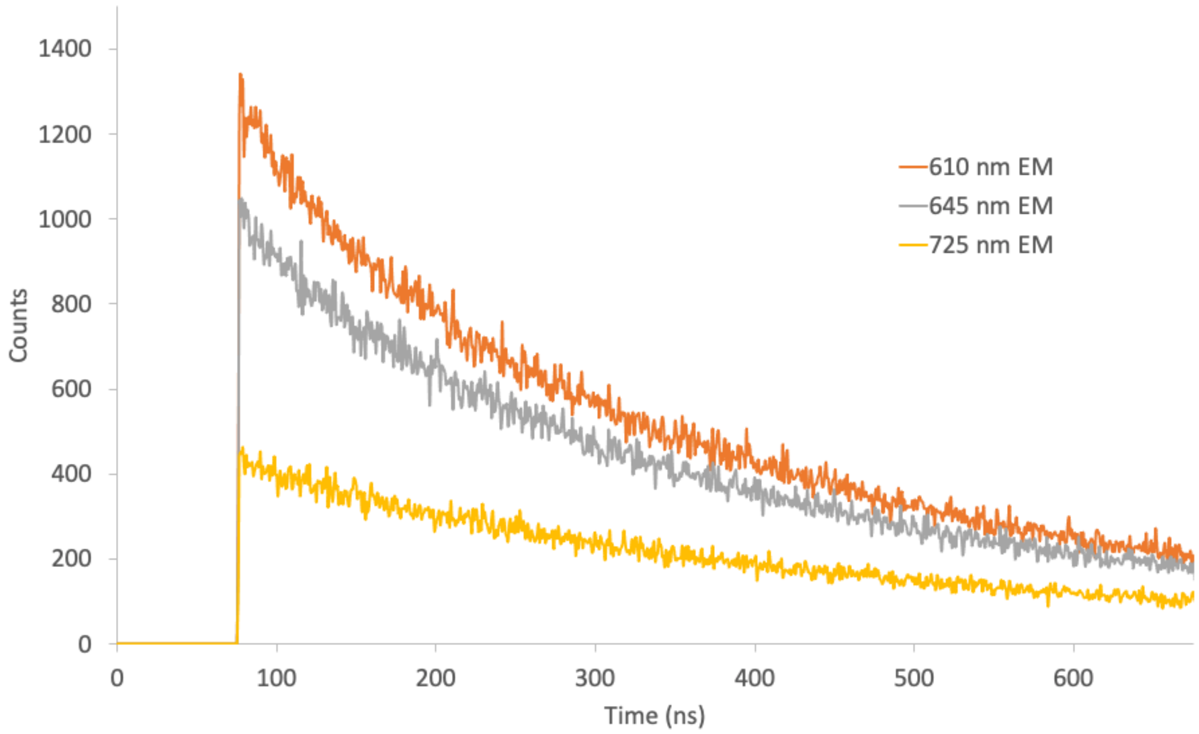

Figure S34. TCSPC decay curve for Pt(II)MTh in the solid state excited at 450 nm.

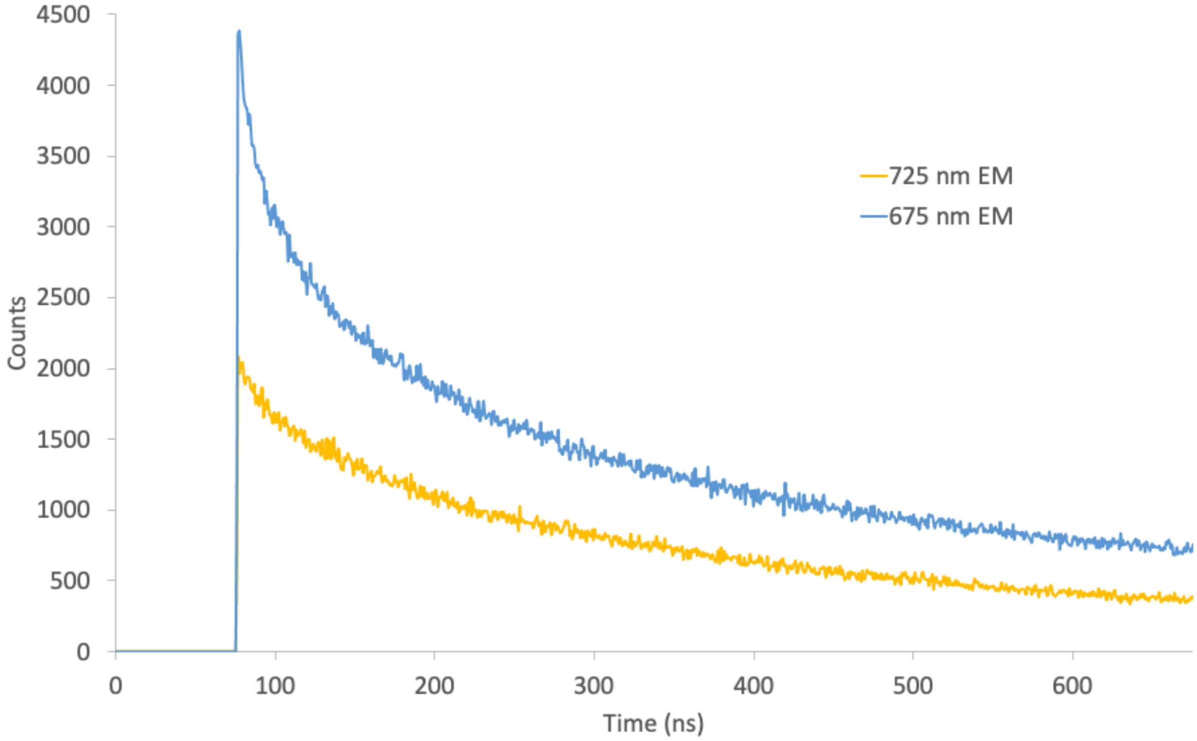

Figure S35. TCSPC decay curve for Pt(IV)DTh in the solid state excited at 450 nm.

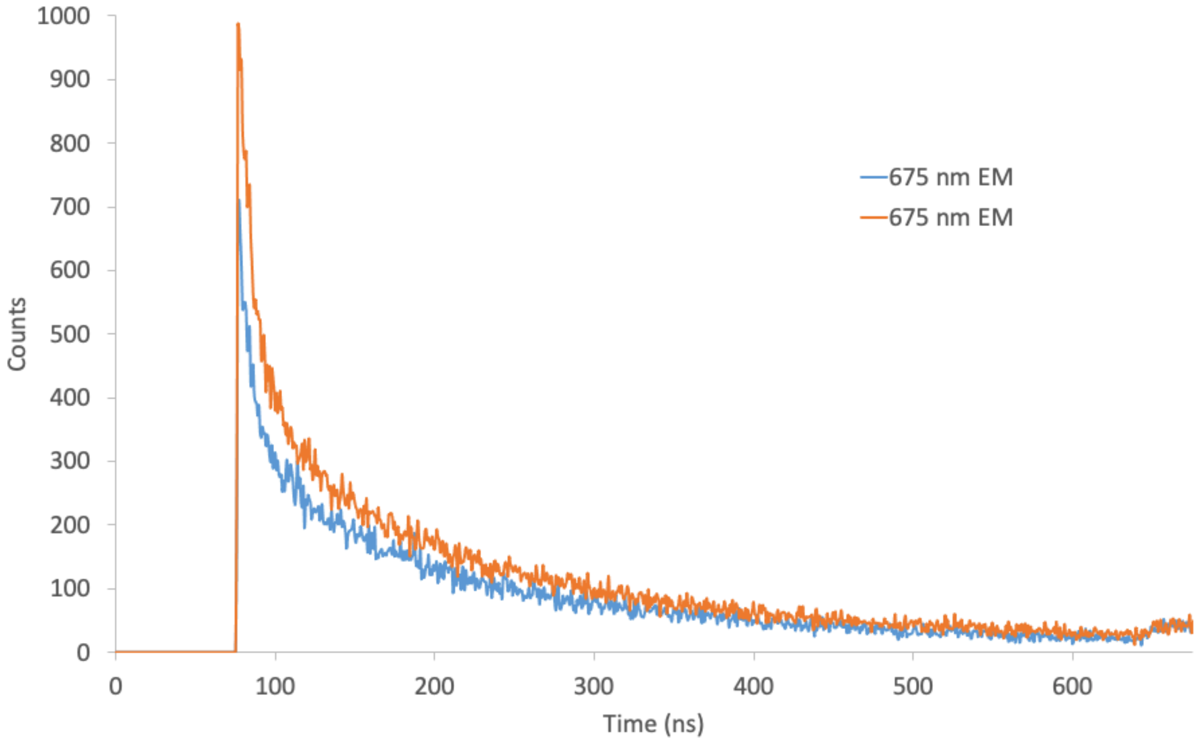

Figure S36. TCSPC decay curve for Pt(II)MPh in the solid state excited at 405 nm.

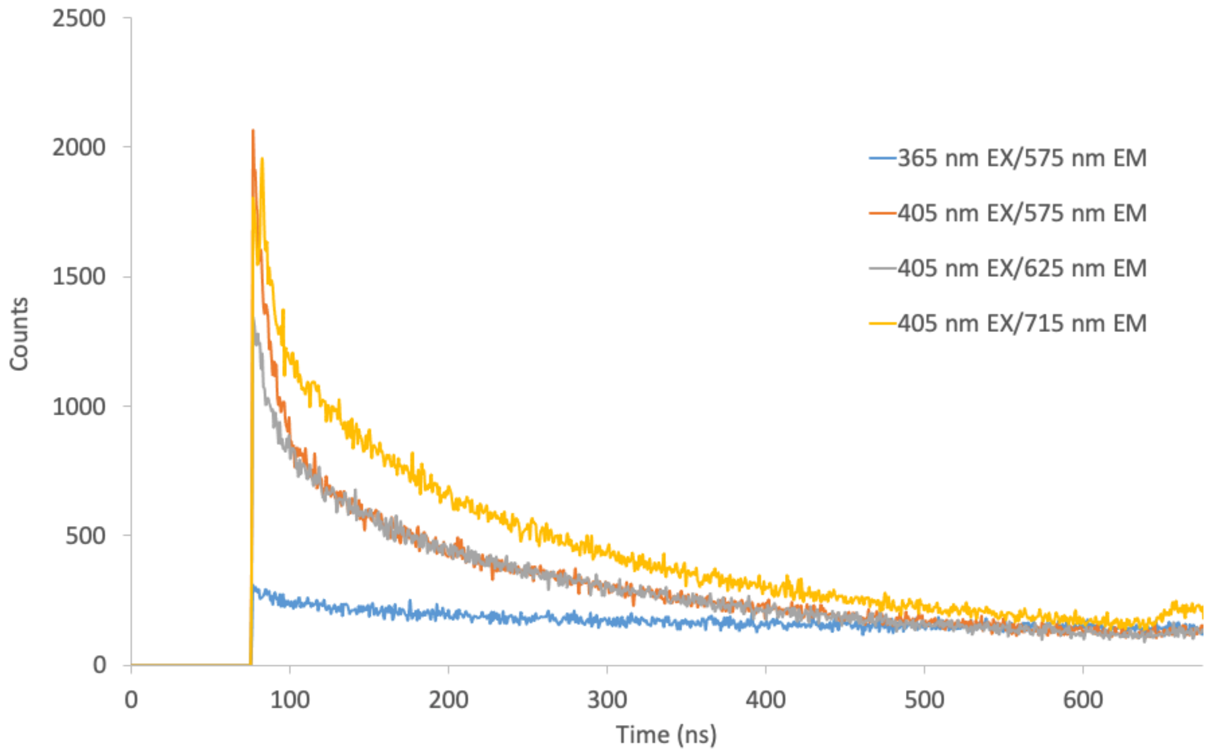

Figure S37. TCSPC decay curve for Pt(IV)DPh in the solid state excited at 365 nm and 405 nm.

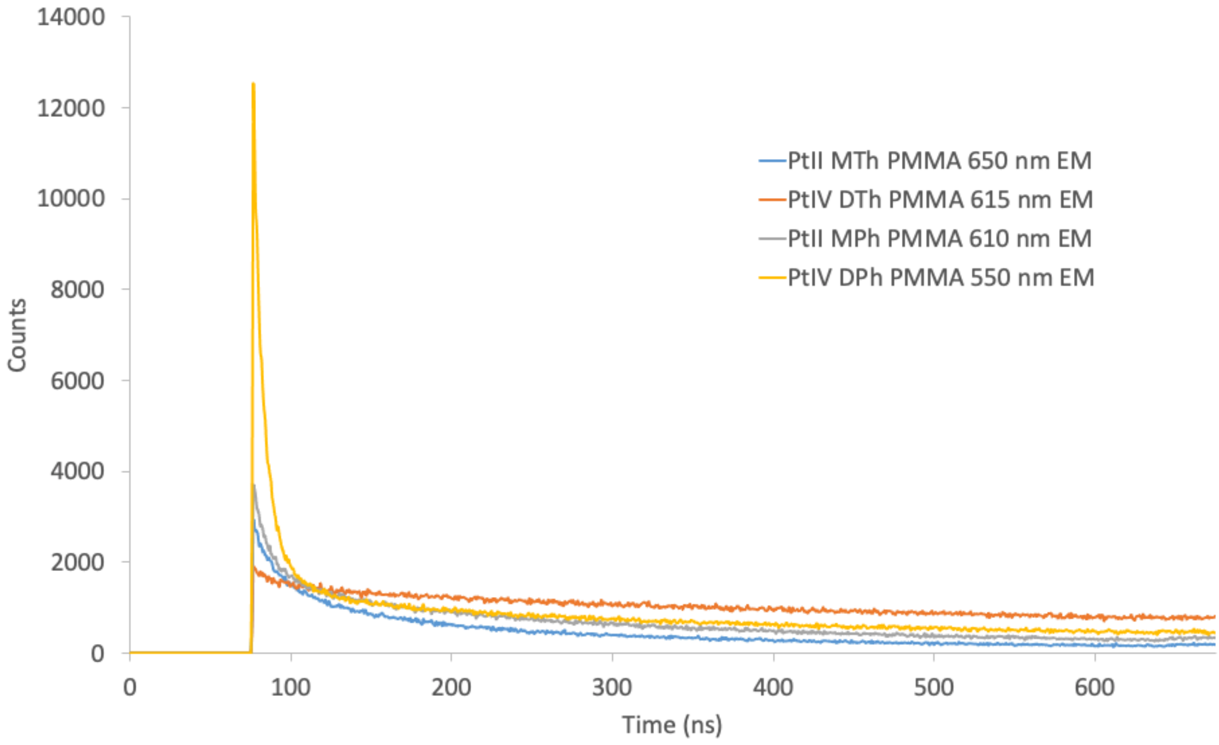

Figure S38. TCSPC decay curves for PMMA films excited at 405 nm.

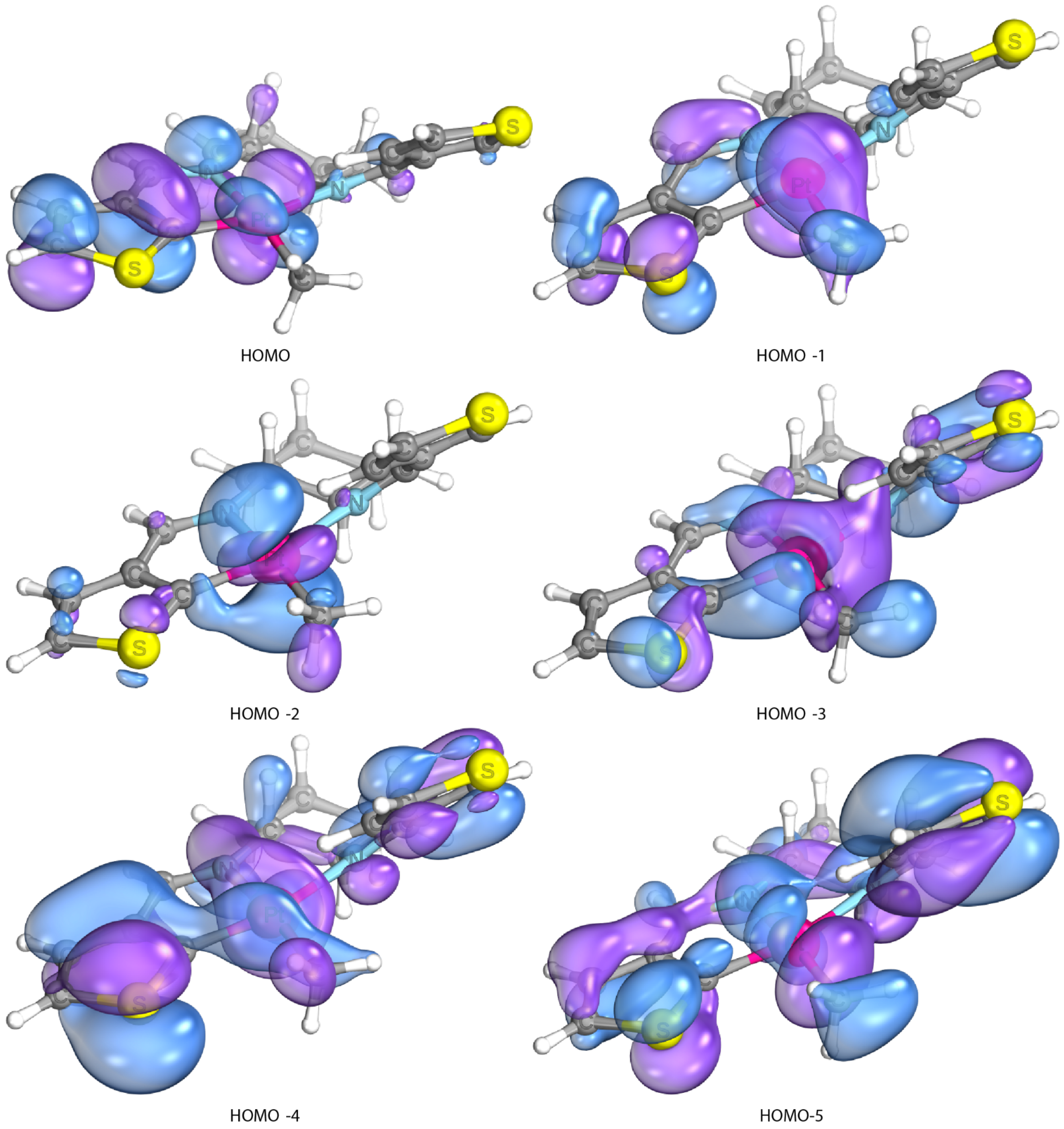

Figure S39: DFT calculated HOMO and near-HOMOs for **PtIIMTh**

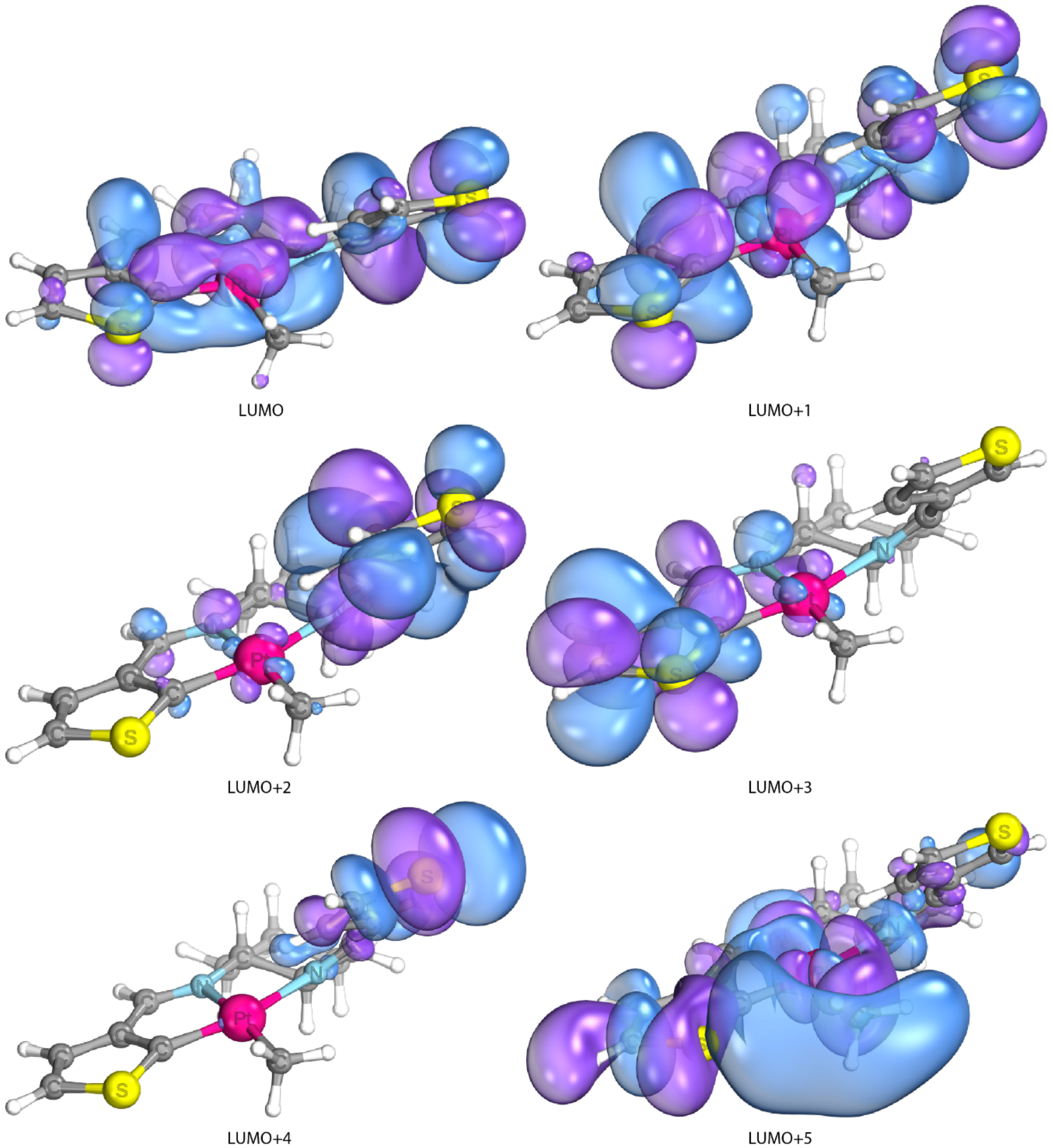

Figure S40. DFT calculated LUMO and near-LUMOs for **PtIIMTh**

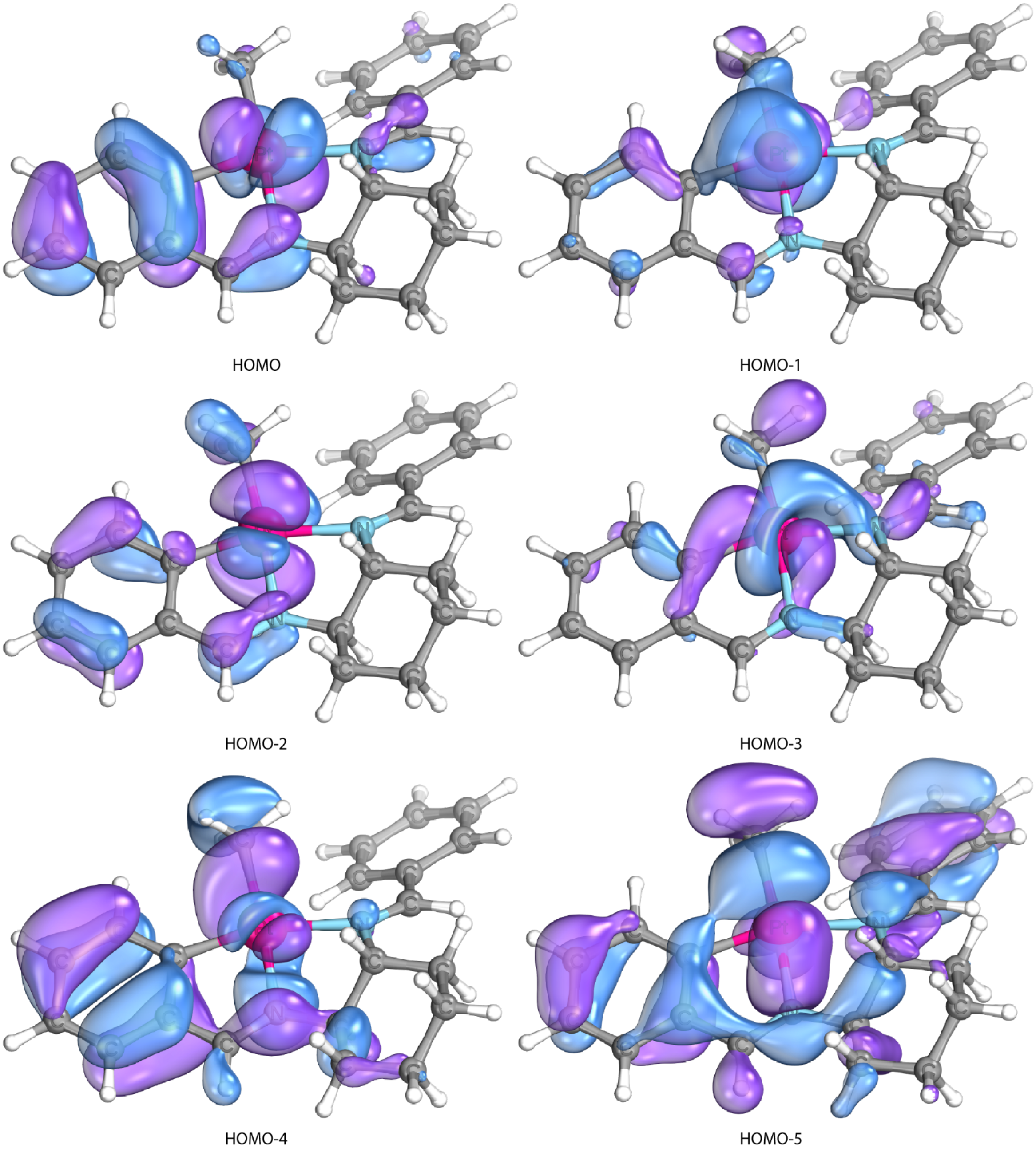

Figure S41. DFT calculated HOMO and near-HOMOs for **PtIIMPh**

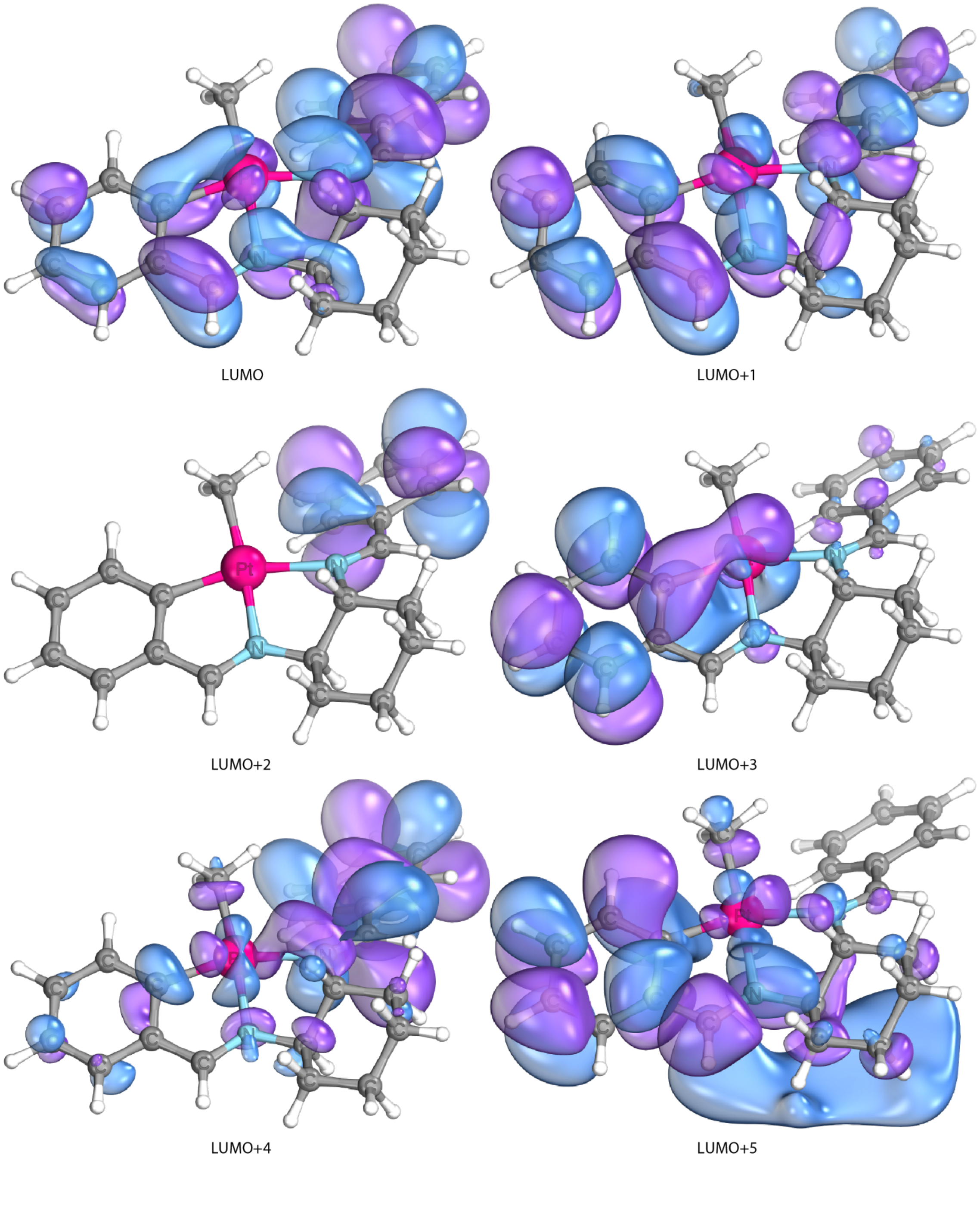

Figure S42. DFT calculated LUMO and near-LUMOs for **PtIIMPh**

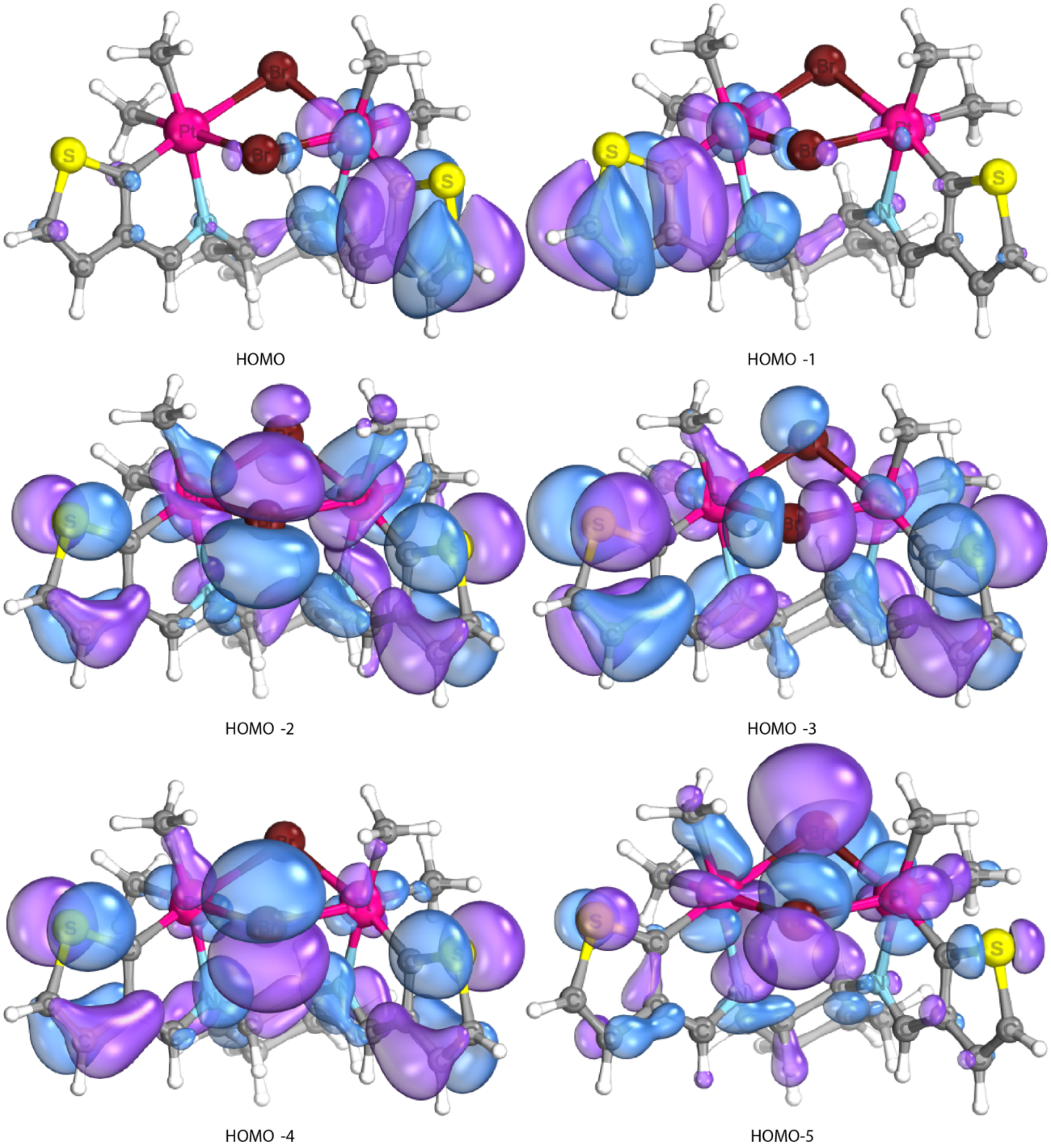

Figure S43. DFT calculated HOMO and near-HOMOs for **PtIVDTh**

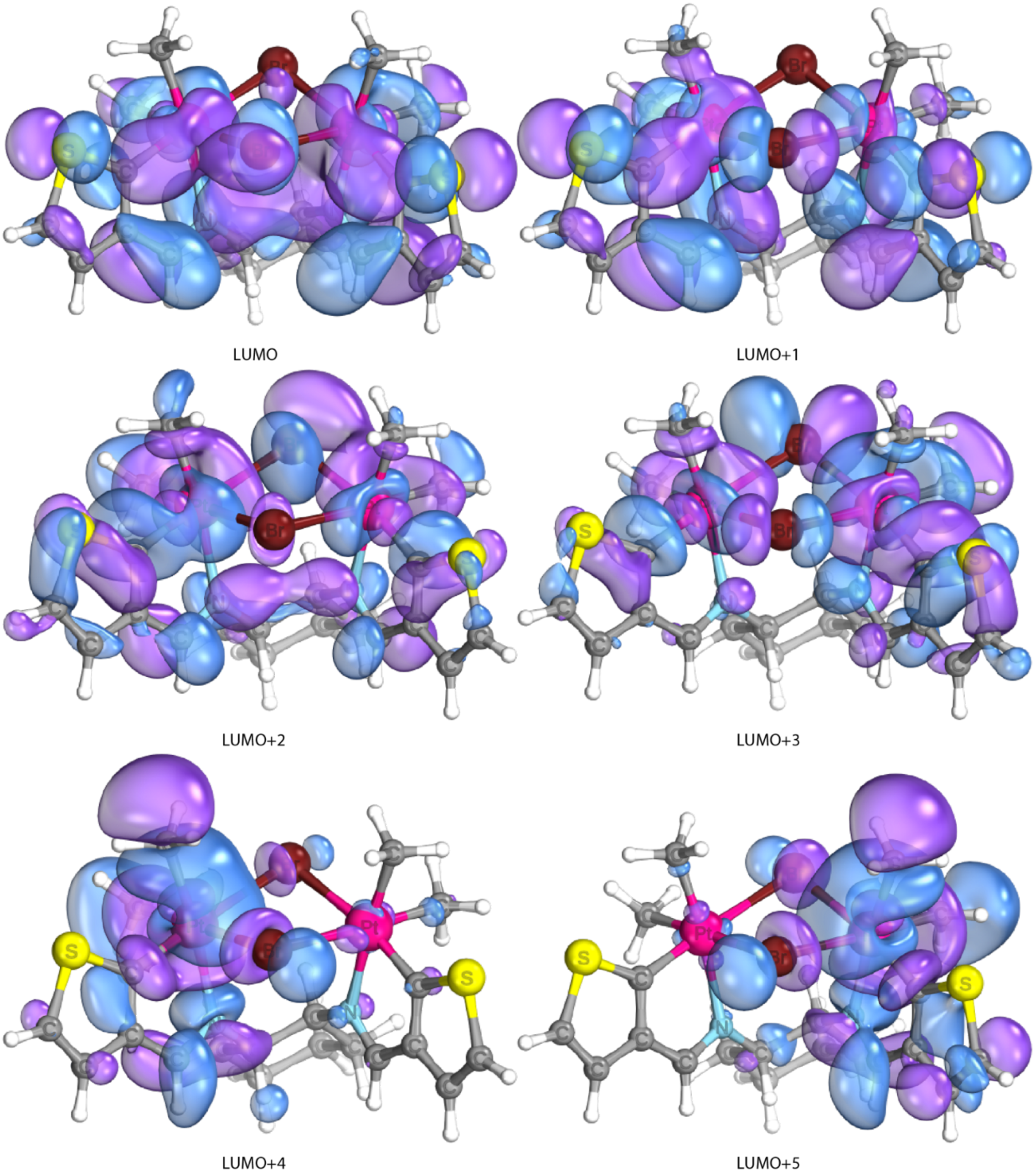

Figure S44. DFT calculated LUMO and near-LUMOs for **PtIVDTh**

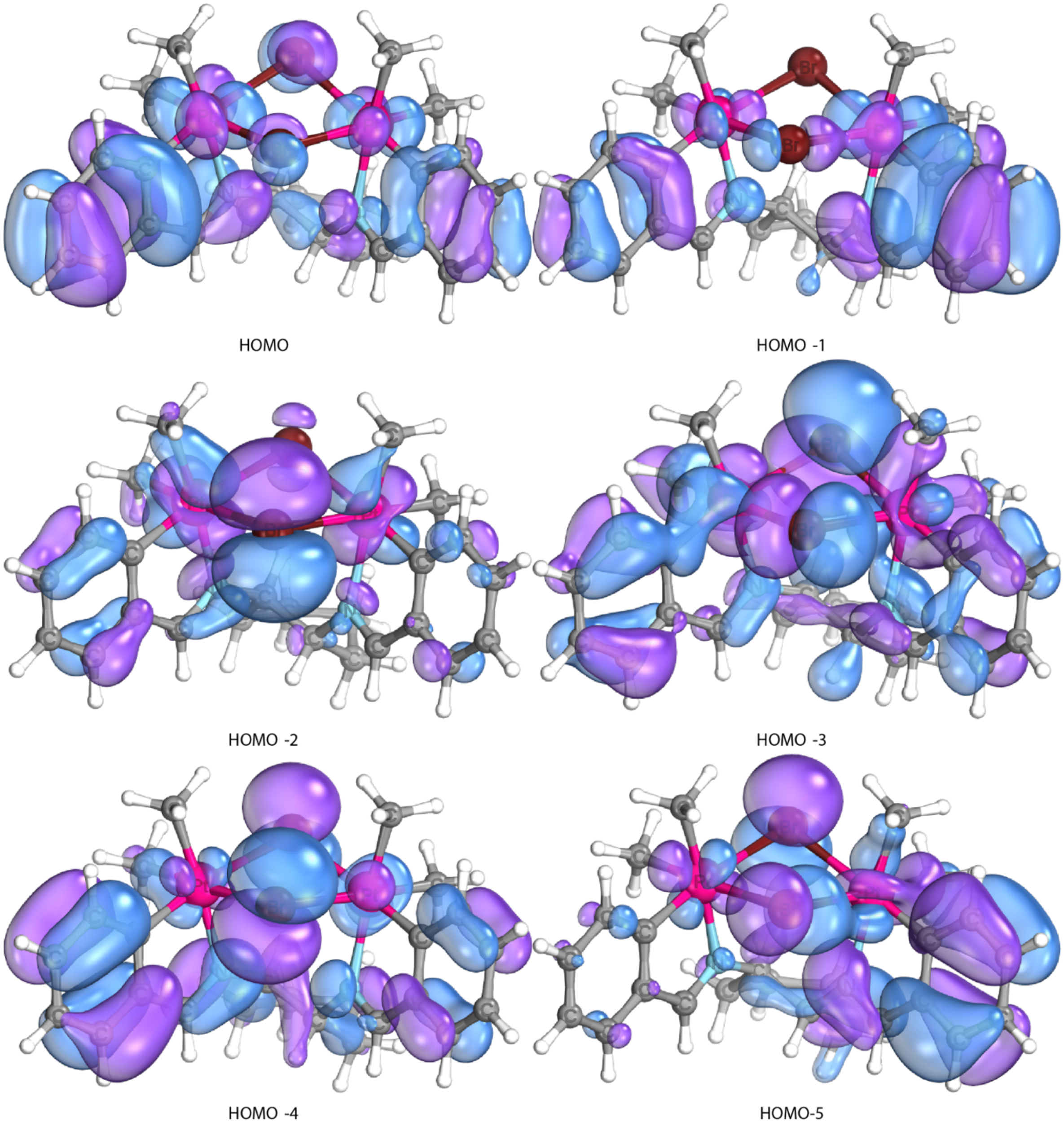

Figure S45. DFT calculated HOMO and near-HOMOs for **PtIVDPh**

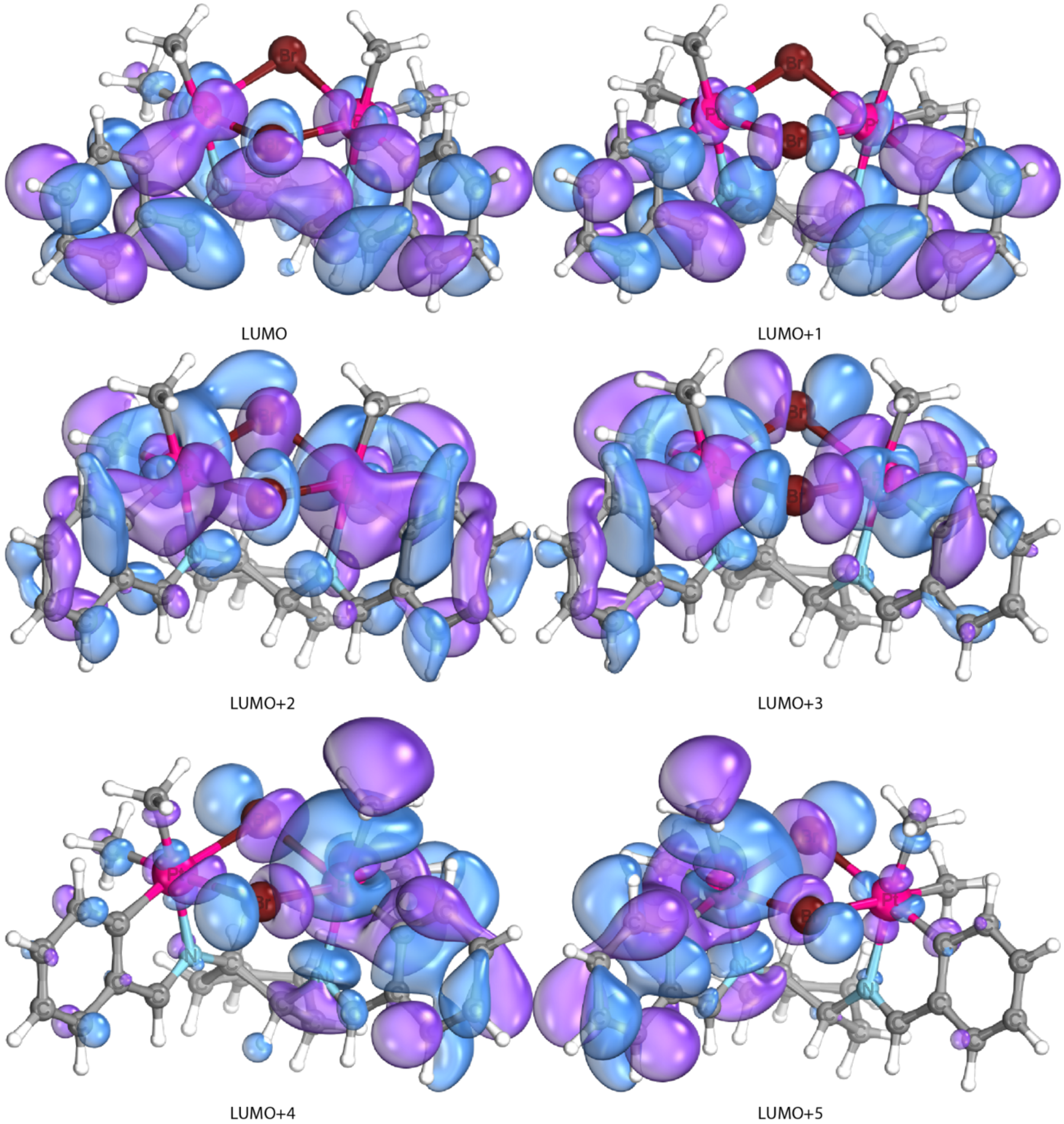

Figure S46. DFT calculated LUMO and near-LUMOs for **PtIVDPh**

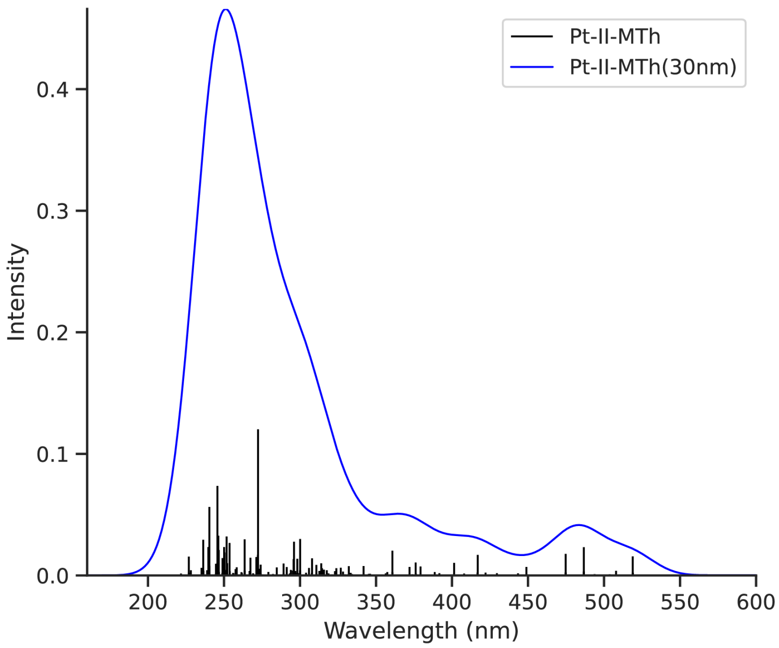

Figure S47. TD-DFT excitation energies and oscillator strengths and visualization with 30 nm linewidth for **PtIIMTh**

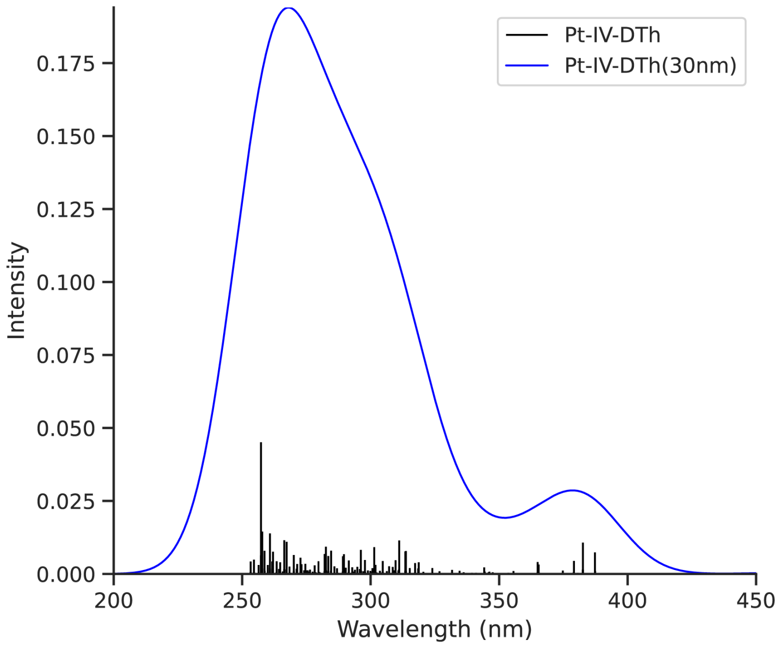

Figure S48. TD-DFT excitation energies and oscillator strengths and visualization with 30 nm linewidth for **PtIVDTh**

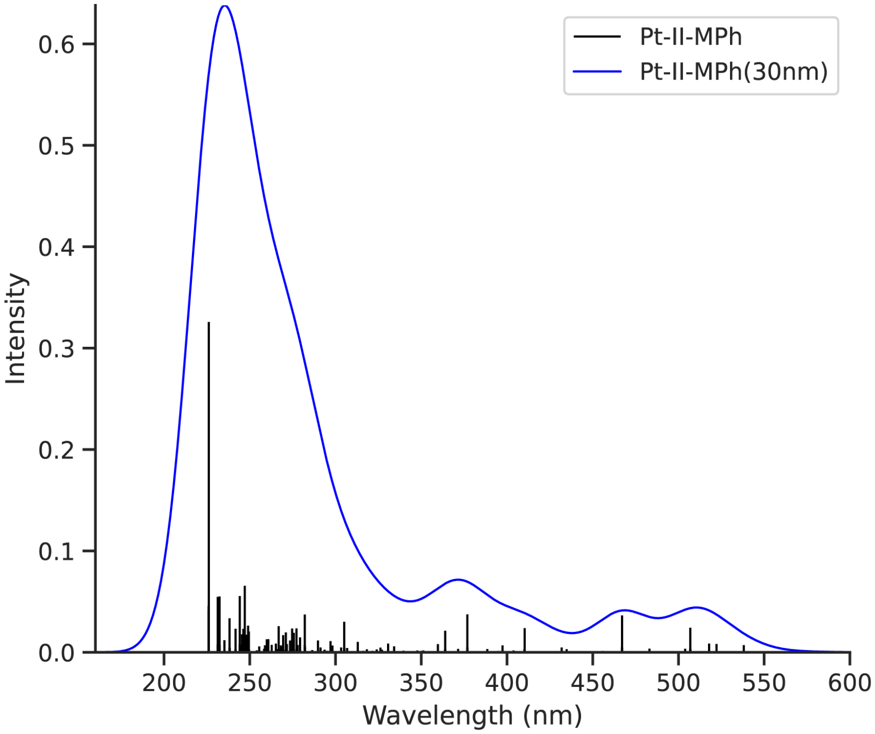

Figure S49. TD-DFT excitation energies and oscillator strengths and visualization with 30 nm linewidth for **PtIIMPh**

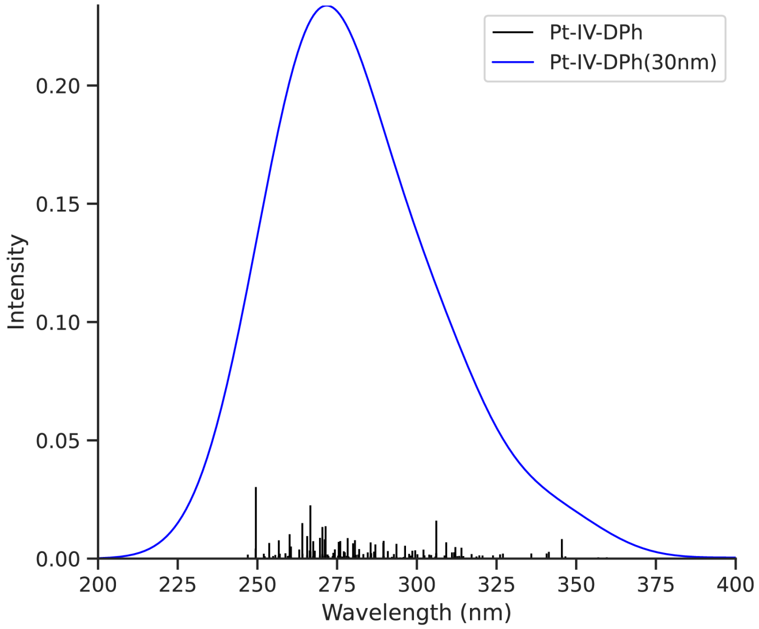

Figure S50. TD-DFT excitation energies and oscillator strengths and visualization with 30 nm linewidth for **PtIVDPh**

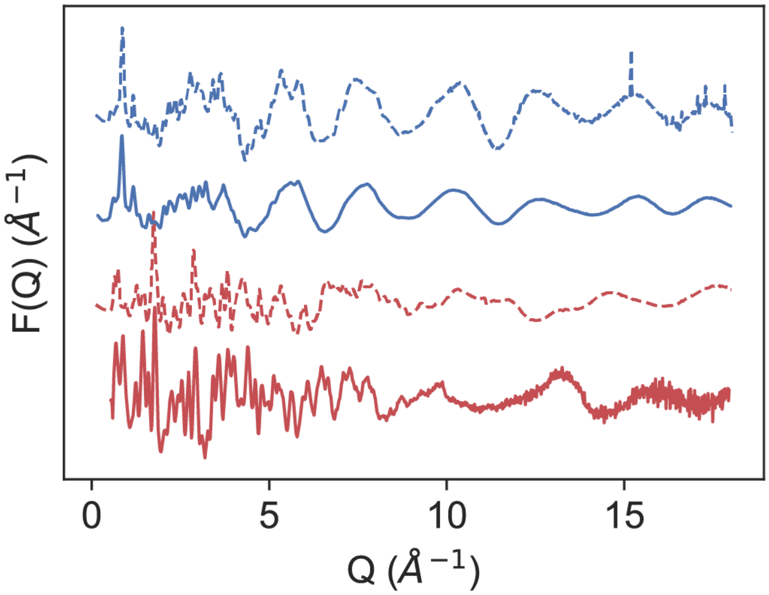

Figure S51. Reduced total scattering structure function $F(Q)$ for **PtIIMTh** (red), **PtIIMPh** (dashed red), **PtIVDTh** (blue), **PtIVDTh** (dashed blue)

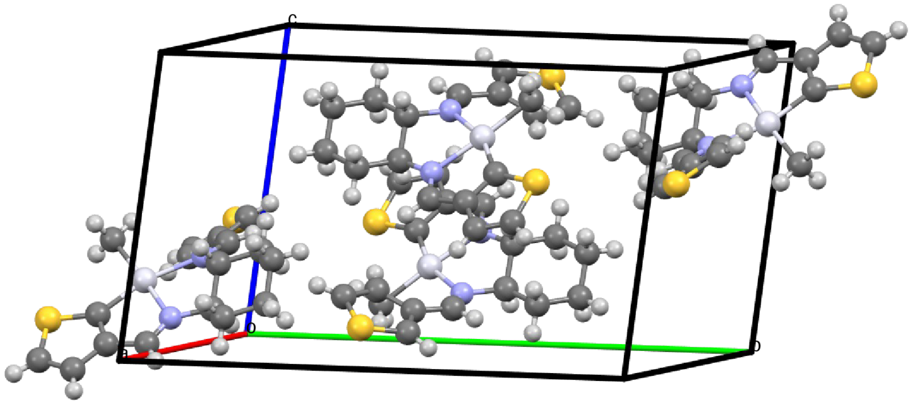

Figure S52. Packing of **PtIIMTh** showing local intermolecular arrangements in solid state

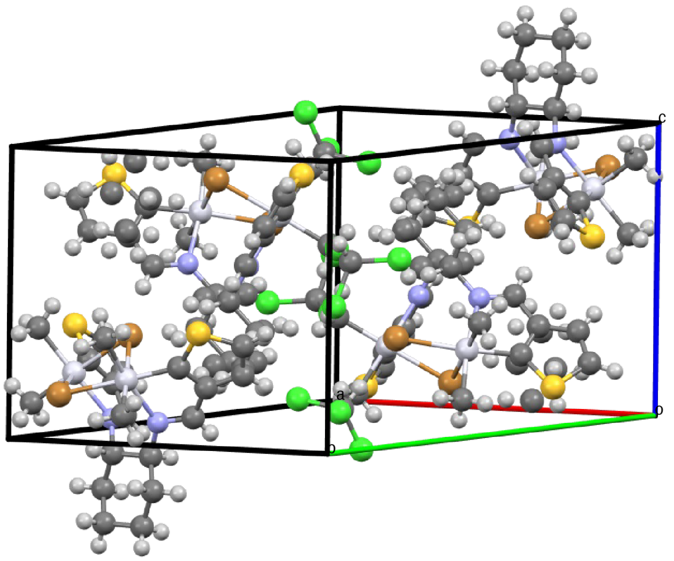

Figure S53. Packing of **PtIVDTh** showing local intermolecular arrangements in the solid state

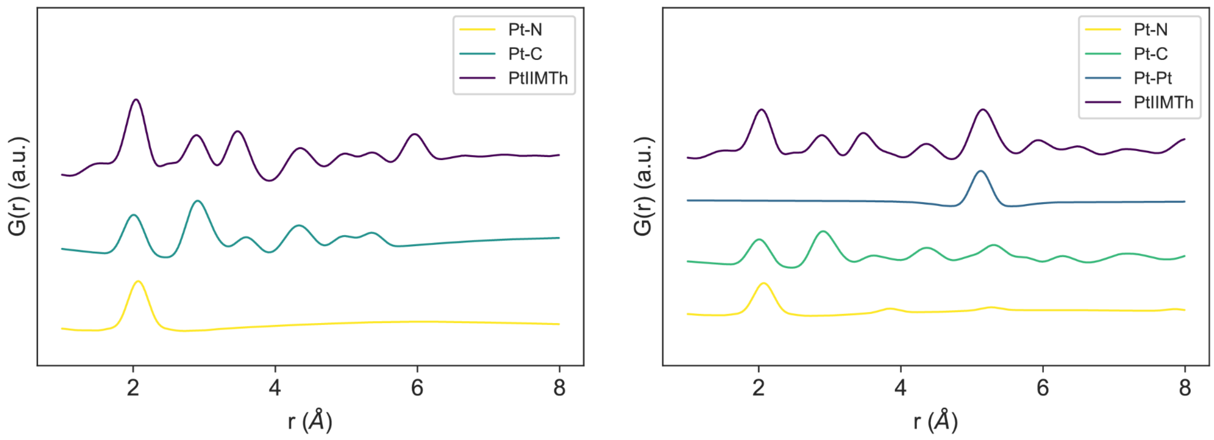

Figure S54. DiffPy calculated total PDF and selected partial PDFs for **PtIIMTh** for both single molecule (left) and discrete crystal packing (right) as is shown in Figure S41.

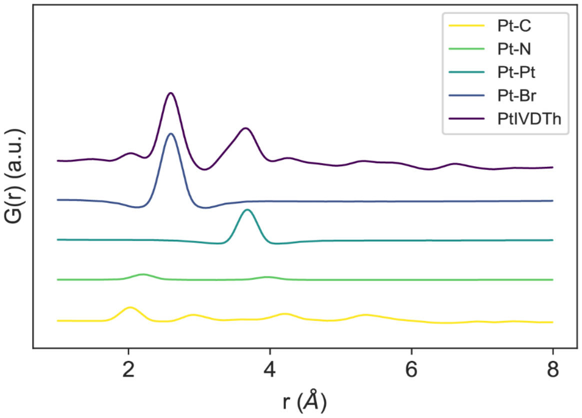

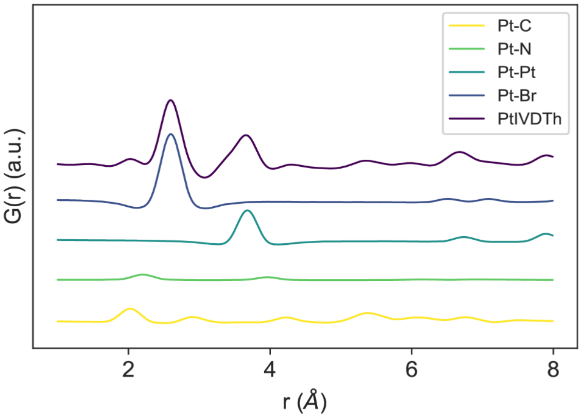

Figure S55: DiffPy calculated total PDF and selected partial PDFs for **PtIVDTh** for both single molecule (left) and discrete crystal packing (right) as is shown in Figure S42. The chloroform solvents of crystallization were manually removed for purposes of the simulation.

PDF Model Description

The calculated pair distribution function, G_{calc}, shown in Figure 8. is treated as a linear combination of intermolecular and intramolecular phase components of **PtIIMTh** using our single crystal structure as a periodic structural model in both cases. This allows separate intramolecular and intermolecular structural parameters to be refined which is known to be necessary to account for the real space pair distribution functions of molecular solids.[1-2]

$$G_{calc} = G_{intra(1.9\,Å-4.7Å\,)} + G_{inter(1.9\,Å-40.0Å\,)} - G_{inter(1.9\,Å-4.7Å\,)}$$

The choice of cutoffs for these distance ranges was determined by examining the width of the correlations which noticeably broaden past 4.7Å. This choice is consistent with the solid-state structure as the Pt-Pt intermolecular separation is 5.12 Å in the single crystal structure which contributes prominently to the PDF as is shown in Figure S43. Parameters are refined in PDFGui over the real space distance range $r = 1.9$ Å $-$ 40.0 Å. The goodness of fit $rw = 0.32$. The Q_{damp} and Q_{broad} experimental resolution factors were determined based on fitting a crystalline Ni beamline calibration standard. Isotropic atomic displacement parameters are refined for non-H atoms, in addition to lattice parameters, a low r quadratic peak sharpening empirical factor for correlated atomic motion[3], scale factors, and a spherical envelope function diameter for finite crystal size.[4] The H atoms U_{iso} are not refined but fixed at 0.035 Å^2 due to the small contribution of H to the overall X-ray PDF. Similarly, the N, S, C atoms U_{iso} for the intermolecular distance ranges are refined as one common parameter for all the intermolecular ligand atom ADPs. Attempts to refine these lighter atoms independently led to unphysically large values for N and S with very little improvement to the model agreement. Refined parameters are collected in Table S4 below.

Parameters	Intramolecular ($r = 1.9 - 4.7$Å)	Intermolecular ($r = 4.7 - 40.0$Å)
a (Å)	8.487	9.083
b (Å)	18.792	18.981
c (Å)	11.058	10.742
δ_2 (Å^2)	3.76	3.76
Pt U_{iso} (Å^2)	0.0133	0.0182
S U_{iso} (Å^2)	0.0127	0.0813
N U_{iso} (Å^2)	0.00446	0.0813
C U_{iso} (Å^2)	0.0109	0.0813
Spdiameter (Å)	152.7	152.7

Table S4. Refined structural parameters for PDFGui fit **PtIIMTh**

References

(1) Prill, D.; Juhás, P.; Schmidt, M. U.; Billinge, S. J. L. Modelling Pair Distribution Functions (PDFs) of Organic Compounds: Describing Both Intra- and Intermolecular Correlation Functions in Calculated PDFs. *J Appl Crystallogr* **2015**, *48* (1), 171–178. https://doi.org/10.1107/S1600576714026454.

(2) Rademacher, N.; Daemen, L. L.; Chronister, E. L.; Proffen, T. Pair Distribution Function Analysis of Molecular Compounds: Significance and Modeling Approach Discussed Using the Example of *p* -Terphenyl. *J Appl Crystallogr* **2012**, *45* (3), 482–488. https://doi.org/10.1107/S0021889812016159.

(3) Jeong, I.-K.; Proffen, T.; Mohiuddin-Jacobs, F.; Billinge, S. J. L. Measuring Correlated Atomic Motion Using X-Ray Diffraction. *J Phys Chem A* **1999**, *103* (7), 921–924. https://doi.org/10.1021/jp9836978.

(4) Kodama, K.; Iikubo, S.; Taguchi, T.; Shamoto, S. Finite Size Effects of Nanoparticles on the Atomic Pair Distribution Functions. *Acta Crystallogr A* **2006**, *62* (6), 444–453. https://doi.org/10.1107/S0108767306034635.
